# Uncovering the architecture of production-driven introgression in Cinisara cattle breed

**DOI:** 10.1186/s12863-025-01337-y

**Published:** 2025-07-11

**Authors:** Viviana Floridia, Arianna Bionda, Katherine Daniela Arias, Annalisa Amato, Matteo Cortellari, Enrico D’Alessandro, Felix Goyache, Vincenzo Lopreiato, Paola Crepaldi, Luigi Liotta, Mario Barbato

**Affiliations:** 1 Department of Veterinary Sciences, Università degli Studi di Messina, Viale Giovanni Palatucci, Messina, 98168 Italy; 2 Department of Agricultural and Environmental Sciences - Production, Landscape, Agroenergy, University of Milan, Via Celoria 2, Milan, 20133 Italy; 3 Animal Genetics and Reproduction Area, SERIDA-Deva, Camino de Rioseco 1225, Gijón, 33394 Spain

**Keywords:** Local breeds, Cattle, *Bos taurus*, Local ancestry inference, Introgression

## Abstract

**Background:**

Local livestock breeds play a pivotal role in maintaining agricultural sustainability, conserving biodiversity, and preserving cultural heritage. These breeds often possess unique genetic characteristics tailored to their specific environments. The Cinisara is a dual-purpose local cattle breed of Podolian origin, primarily farmed in western Sicily, Italy. However, reports of spurious crossbreeding with cosmopolitan breeds aimed at improving the breed productivity exist. To assess the conservation status and ongoing selective pressures on this unique breed, we genotyped 71 unrelated Cinisara cattle (CIN_A) at 65k SNPs, and extended the dataset with publicly available genotype data of 30 Cinisara individuals sampled 20 years ago (CIN_B). We also included 194 individuals from seven cattle breeds, including the Podolica (POD) breed and the cosmopolitan Holstein (HOL) and Brown Swiss (BRW) breeds. We assessed the genetic diversity, population structure, and determined the extent of introgression from cosmopolitan breeds into Cinisara using local ancestry inference.

**Results:**

Population structure analyses confirmed the Cinisara’s Podolian lineage and revealed significant HOL and BRW introgression. While both Cinisara populations, CIN_A and CIN_B, displayed broadly comparable genetic diversity to larger breeds, CIN_B showed reduced heterozygosity and increased inbreeding. CIN_A exhibited higher introgression, suggesting ongoing crossbreeding. Local ancestry was inferred using POD, HOL, and BRW references. CIN_A showed about 258/257 HOL/BRW introgressed SNPs, intercepting 186/131 genes and 1,584/1,772 QTLs. CIN_B had approximately 256/254 HOL/BRW introgressed SNPs, intercepting 218/184 genes and 547/437 QTLs. Predominantly, these regions overlapped with milk production QTLs, but some intercepted genes linked to unique Cinisara traits, like milk quality and climate adaptation, potentially altering breed typicality. Notably, CIN_B shows a potentially higher relative BRW contribution, while CIN_A shows a higher HOL contribution.

**Conclusion:**

Our findings align with the reports of crossbreeding with cosmopolitan breeds to enhance the production performance of Cinisara, and reflect breeding choices such as a reduction in BRW crossing or a preference for HOL. This raises significant concerns regarding the preservation of local breeds, livestock biodiversity, and their cultural and economic value, and highlights the importance of developing informed breeding strategies that balance production improvements with the conservation of genetic heritage.

**Supplementary Information:**

The online version contains supplementary material available at 10.1186/s12863-025-01337-y.

## Introduction

The global human population is projected to reach approximately nine billion by 2050, driving a significant increase in the demand for animal-derived food and placing greater pressure on livestock production systems. In response, animal husbandry practices have become more intensified, prioritising high-yielding breeds that maximise production efficiency [1]. However, this shift has contributed to the decline of local breeds, which are typically raised through traditional farming methods [2, 3].

Local livestock populations encapsulate the history, culture and traditional agricultural systems of their territories [4–7]. In addition, they play crucial social and economic roles in marginal or inner areas. Local breeds have adapted to the territories they inhabit and their climatic condition exhibiting adaptive traits, including improved heat dissipation, metabolic flexibility, and nutritional efficiency [5–9]. Moreover, their limited exposure to intense production-driven selection has allowed them to retain high levels of standing genetic variation [10], essential for addressing global challenges. As the breeding of cosmopolitan livestock breeds becomes increasingly difficult due to their susceptibility to environmental and physiological stressors [11–14], such as reported heat stress in dairy cattle [15–17], the conservation of resilient populations has become a pressing priority [3, 7, 18].

Sicily, Italy’s largest island, hosts several local breeds, including the Cinisara cattle. The Cinisara is a dual-purpose autochthonous breed. According to the Banca Dati Nazionale (BDN), there are approximately 6,775 registered animals (BDN; accessed on 03/03/2025, www.vetinfo.it/). However, the official herdbook recognizes 5,311 animals as belonging to the breed (ANARB; accessed on 10/06/2025, www.anarb.it/portfolio/assemblea-generale-anarb-2025/). The Cinisara is a Podolian lineage-derived cattle breed [19], introduced to Sicily in 1860 from Calabria (the southernmost region of mainland Italy) to restock the Sicilian cattle population, which had been devastated by a severe epidemic affecting herds [20]. However, the genetic origins of Cinisara remain incompletely understood, with some historical sources suggesting a possible relationship with Pinzaguer [20]. Until 1985, Cinisara was included in the herdbook of the Modicana breed. Between 1985 and 1995 Cinisara was reclassified as a ‘population with limited diffusion’, until officially recognized as a distinct breed in 1995.

Currently, Cinisara is reared on the western coast of Sicily, particularly in the territory of Cinisi, a predominantly mountainous area characterised by warm and sultry summers. This region provides grazing lands with indigenous grasses, which impart unique qualities to traditional Cinisara cheeses, such as ‘caciocavallo palermitano’ [21–23]. Although not comparable with the productivity of highly specialised dairy breeds, the Cinisara cattle can produce 3,700 kg of milk per lactation. This milk is notable for its high protein (3.5%) and fat (3.6%) content, making it particularly suitable for cheese [24]. Furthermore, Cinisara milk is rich in polyphenols, terpenes, unsaturated fatty acids (Omega 3 and 6) and antioxidants [24]. Furthermore, Cinisara is a source of meat quality products such as salami and bresaola [25–27], otherwise recognised for their richness in vitamins and conjugated linoleic acid, and low cholesterol content.

To conserve the Cinisara cattle breed, preserve its traditional breeding system within its area of origin, and enhance its productivity, the Cinisara breed Consortium was established in 2005 [28]. However, reports of unregistered crosses between Cinisara and Holstein Friesian and Brown Swiss, aiming at improving the economic profit of Cinisara productivity, exist. Additionally, in the cow-calf line systems typical of Sicilian farming, Limousine bulls are widely used to sire F1 Cinisara calves. These calves are valued for their smaller birth size, which helps reduce dystocia, and are typically raised for slaughter. Here, we apply genomic tools to determine the conservation status of Cinisara, with a special focus on genetic diversity and ongoing selection of this unique population. To this end, we conducted a fine-grained investigation of the introgression levels of cosmopolitan breeds into Cinisara. By analysing these patterns, we aim to support the conservation and sustainable management of this valuable genetic resource, ensuring its role in future agricultural systems.

## Materials and methods

### Biological samples and dataset creations

Peripheral blood samples of 71 Cinisara (CIN_A), 30 Modicana (MOD) and 29 Limousine (LIM) were collected in 2024 by professional veterinary personnel following the recommendations of the European directive 2010/63 and with the permission of the Ethical Committee of Messina University (Authorization number 19_2023). The animals were privately owned and bred by commercial farms in Sicily, with all breeders providing specific declarations consenting to their inclusion in the study. CIN_A was sampled in farms located in the Sicilian province of Palermo, whereas MOD and LIM were sampled in the Sicilian province of Ragusa. The DNA extraction and genotyping were outsourced and the latter performed using the Affymetrix 65k SNPchip. Publicly available genotype data of 30 additional Cinisara individuals born between 1999 and 2010 (CIN_B), 24 Podolica (POD), 32 Italian Holstein Friesian (HOL), 32 Italian Brown Swiss (BRW) and 24 Pinzgau (PNZ), genotyped with the BovineSNP50v2 BeadChip (Illumina Inc.) [29] were included in the dataset for comparison. Finally, we included 23 N’Dama (NDA) individuals as an outgroup [30].

SNPs positions were updated to the ARS-UCD1.2 bovine genome map (https://www.ncbi.nlm.nih.gov/datasets/genome/GCF_002263795.1/) SNP located on sex chromosomes or with unknown map positions were removed. PLINK v1.9 [31] was used to remove individuals with > 10% missing genotypes. Relatedness among individuals within breeds was assessed, and one random individual of each closely related pair of individuals (first-degree relationships: *--king-cutoff 0.117*) was excluded using PLINK v2.0 [31, 32]. This dataset was used for the runs of homozygosity (ROH) analysis [33–35].

To assess population structure, we further pruned the dataset excluding loci with minor allele frequency (MAF) < 0.05 and pruning for high linkage disequilibrium (LD). LD pruning was performed applying the *indep-pairwise* function in PLINK v1.9, removing a random SNP of each pair having *r*^*2*^ > 0.5 from sliding windows of 50 SNPs and a 10 SNPs forward step. To feed local ancestry and haplotype-sharing analyses we performed phasing and imputation of missing genotypes using BEAGLE v5.4 [36] on the dataset filtered for MAF but prior LD pruning.

### Genetic diversity and population structure

Genetic diversity of the population has been estimated through observed heterozygosity (H_o_), computed using KING v2.3.2 [32] and ROH. The minimum number of SNPs to define a ROH and the window size was calculated using the *L* parameter, whereas the density threshold was chosen after calculating ROH coverage for values ranging from the mean density of the analysed markers to 250 kb/SNP [33, 35]. Thus, ROH were analysed using the --homozyg flag implemented in PLINK v1.9 with a sliding window of 47 SNPs (*homozyg-window-snp = 47)* and excluding short ROH originated by linkage disequilibrium. A ROH was called if it was at least 1 Mb long (*homozyg-kb* = 1000) and contained at least 47 consecutive homozygous SNPs (*homozyg-snp* = 47), while the regions with less than one SNP per 129 kb (*homozyg-density* = 129) or a gap of over 500 kb (*homozyg-gap* = 500) between consecutive SNPs were excluded. In addition, no heterozygous SNPs (*homozyg-window-het* = 0) were permitted, whereas two missing genotypes per window were allowed to account for potential genotyping errors (*homozyg-window-missing* = 2). The individual genomic inbreeding coefficient (F_ROH_) was calculated as the ratio of the total length of ROH to the total length of the autosomal genome covered by SNPs [37] and then the F_ROH_ values were partitioned into five ROH length categories: 1–2 Mb, 2–4 Mb, 4–8 Mb, 8–16 Mb, and > 16 Mb. Current effective population size (N_e_) was estimated using SNeP v1.11 [38, 39] with Sved & Feldman’s mutation rate modifier, sample size correction and a permitted maximum distance between SNP pairs of 200 Mb.

To visualise the complex evolutionary relationships among populations, we performed a principal component analysis (PCA) using the ’--pca’ within PLINK v1.9, and a Neighbour-Net using Reynolds’ distances computed with a custom software (https://github.com/barbatom/ReynoldsDist) and plotted using SplitsTree v6.3.30 [40]. A global ancestry analysis was conducted using ADMIXTURE v1.3.0 [41]. We tested *K* theoretical ancestral populations with *K* from 2 to 8 (the latter being the total number of breeds in our study). A cross-validation (CV) error was computed for each *K* and CLUMPAK beta version [42] was used to plot ancestry solutions. The occurrence of gene flow was further investigated using TreeMix v1.13 [43], which evaluates the relationship among the sample populations with their ancestral population and infers *m* gene flow events (visualised as edges). Following the procedure reported in Pickrell et al., 2012 [43], we built a consensus maximum-likelihood tree incorporating multiple migration events (*m* = {1, 8}), with 10 iterations per value of *m*. NDA was used as an outgroup and the block size was set to 2,000 for all the Treemix analyses. The optimal number of migration edges was identified using the 'OptM' v0.1.8 R package [44]. The robustness of the tree topology of the optimal model identified using OptM was assessed generating 500 bootstrap replicates and 30 independent runs. A consensus tree was obtained with PHYLIP v3.697 [45]. Gene flow events among populations were further evaluated using the f3 test implementation provided with TreeMix. We assessed haplotype sharing among individuals by estimating Identity By Descent (IBD) with RefinedIBD v4.1 [46]. Specifically, we set a 10 Mb sliding window and established a threshold for retaining IBD segments at a minimum length of 0.2 Mb and a LOD score of 3.0 or higher. To minimize edge effects, 0.15 Mb were trimmed from the extremes of shared haplotypes. For each breed pair, haplotype sharing was summarized as the median total length of the shared segments across all individual comparisons, with pairs showing no shared haplotypes assigned a value of 0. Haplotype sharing among breeds was plotted using the'circlize' v0.4.16 R package [47]. 

### Local ancestry inference

Local genomic ancestry inference (LAI) was performed using ELAI v1.01 [48], which uses a two-layer hidden Markov model. The upper-layer clusters represent different populations (ancestral reference populations) while the lower-layer clusters represent specific haplotypes (target population). Haplotypes in the reference population are used to define features of each small genomic region in the target populations. To perform the local genomic ancestry analyses, three reference populations were used: POD as a representative of the ancestral Podolian background, and HOL and BRW that were identified by the former gene flow analyses as candidate sources of introgression. Each Cinisara population (CIN_A and CIN_B) was individually tested as target population. Analyses were performed using the following parameters: 20 expectation maximisation steps (*-s*), 3 upper clusters (*-C*), 15 lower clusters (*-c*) and 50 previous generations before the admixture event (*-mg*). Those SNPs that either were not identified in a given population (*--exclude-miss1*) or for which the position was not recorded in the SNP position file (*--exclude-nopos*) were excluded from the analysis.

The introgression proportions of each reference population (HOL, BRW, POD) were identified in both Cinisara populations. SNPs within the 99^th^ percentile of local introgression proportion for each reference were considered highly introgressed. An ideogram illustrating the distribution of highly introgressed regions (99^th^ percentile) in the Cinisara groups was created using the ‘Rideogram’ v0.2.2 R package [49]. Based on the results of HOL and BRW introgression, we further investigated the regions spanning 20 kb upstream and downstream of each highly introgressed SNP. To identify the genes associated with these regions, we compared them against the ARS-UCD1.2 annotation (https://www.ncbi.nlm.nih.gov/datasets/genome/GCF_002263795.1/) using Bedtools v2.30.0 [50]. Additionally, Bedtools was also used to find intersections between these genomic regions and known quantitative trait loci (QTLs) in bovine species, publicly available at the AnimalQTLdb website [51] (accessed on 28 January 2025).

## Results

### Genetic diversity and population structure

After quality control we retained 25,880 SNPs and 294 individuals; filtering for relatedness left 196 individuals from eight breeds (Table 1). Filtering for MAF left 25,279 SNPs and LD-pruning further reduced the loci count to 23,473. Before imputation, the genotyping rate was 96%.

H_o_ ranged from 0.306 to 0.359 for MOD and CIN_A, respectively (Table 1). Cinisara populations showed relatively low F_ROH_ values. CIN_B displayed significantly higher average F_ROH_ values compared to CIN_A (0.042 vs. 0.017, respectively), but also a higher within-population variability, with some individuals showing inbreeding close to 0.2. Moreover, the F_ROH_ distribution across various ROH length classes was more balanced in CIN_B than in CIN_A, where longer ROH segments were less represented (Table 1 and Additional File 1). Effective population size values ranged from 24 for LIM to 228 for CIN_A, with N_e_ = 170 for CIN_B (Table 1).

**Table 1:** Sample information and diversity statistics. Sample size before (N) and after (N_QC_) quality checks, observed heterozygosity (H_o_), inbreeding (F_ROH_), and current effective population size (N_e_) are provided

Breed	Acronym	Source	*N* (*N*_QC_)	H_O_ (SD)	F_ROH_ (SD)	*N*_e_
Brown Swiss	BRW	[29]	32 (23)	0.315 (0.01)	0.093 (0.03)	112
Cinisara	CIN_A	This study	71 (43)	0.359 (0.01)	0.017 (0.03)	228
	CIN_B	[52]	30 (24)	0.348 (0.03)	0.042 (0.06)	170
Holstein	HOL	[29]	32 (23)	0.352 (0.01)	0.060 (0.03)	108
Limousine	LIM	This study	29 (26)	0.347 (0.01)	0.020 (0.01)	24
Modicana	MOD	This study	30 (15)	0.306 (0.02)	0.087 (0.05)	46
N’Dama	NDA	[30]	23 (21)	0.239 (0.01)	0.014 (0.02)	469
Pinzgau	PNZ	[29]	24 (19)	0.356 (0.01)	0.042 (0.02)	104
Podolian	POD	[29]	24 (22)	0.348 (0.03)	0.040 (0.06)	219

**Fig. 1:**
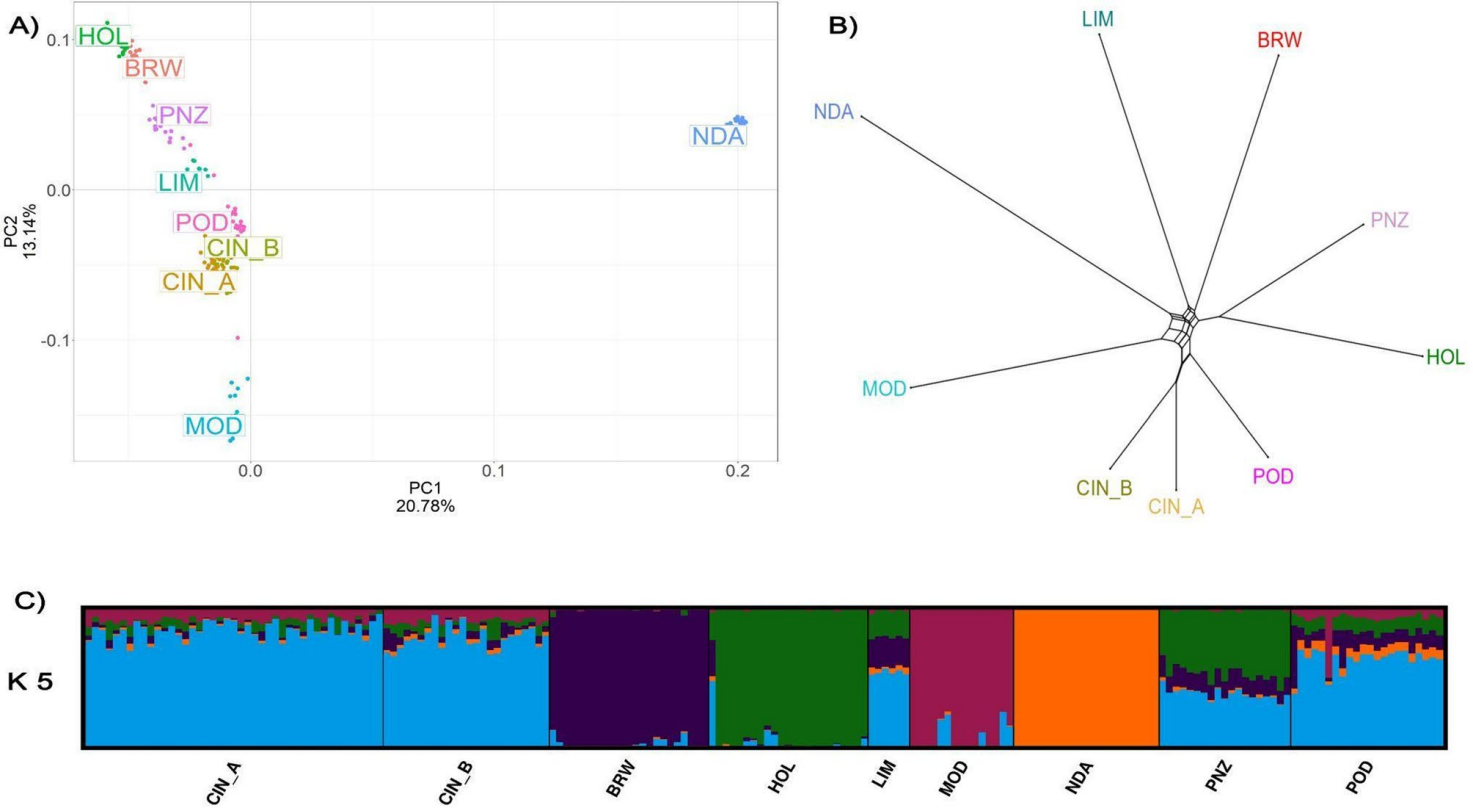
Genetic diversity and population structure. (**A**) Graphic representation of the first two principal components (PC) from a principal component analysis (PCA). PC1 separated NDA from others and clustered together the breeds of southern Italian origin (CIN_A, CIN_B, POD, MOD) and those from the rest of Europe (LIM, BRW, PNZ, HOL); PC2 distinguished between the two cosmopolitan dairy breeds (HOL and BRW), the dual-purpose (PNZ) and beef (LIM) breeds, the Italian populations of Podolian origin (CIN_A, CIN_B, POD), and the MOD breed. (**B**) Neighbour-Net graph based on Reynolds’ distances. (**C**) Proportion of each sample assigned to *K* = 5 genetic clusters using ADMIXTURE. Breed labels are available in Table 1

In the PCA, the first principal component (PC), accounting for 20.78% of the total variance, clearly separated NDA from other populations, while grouping together breeds of southern Italian origin (CIN_A, CIN_B, POD, MOD), and those from the rest of Europe (LIM, BRW, PNZ, HOL; Fig. 1A). The second PC (13.14%) discriminated the cosmopolitan dairy breeds (HOL and BRW), PNZ and LIM against the Italian populations of Podolian origin (CIN_A, CIN_B, POD), and MOD (Fig. 1A). This differentiation was more evident when the PCA was performed excluding NDA (Additional File 2). Neighbour-Net analysis was highly consistent with PCA (Fig. 1B), clustering the two groups of Cinisara together with POD, and near MOD. In the Admixture analysis, the CV test identified *K* = 5 as the best clustering solution. From *K* = 2 to 4, we observed the separation of NDA, BRW, and HOL clusters, respectively. At *K* = 5 a private cluster for MOD emerged, with CIN_A and CIN_B sharing the same ancestry component prevailing in POD, and showing introgression sourcing from the MOD cluster and, to a lesser extent, HOL and BRW. This latter introgression was more evident in CIN_B than CIN_A. A private cluster for the Cinisara breed appeared at *K* = 7, although prevalent only in some CIN_B individuals (Fig. 1C, Additional File 3).

**Fig. 2:**
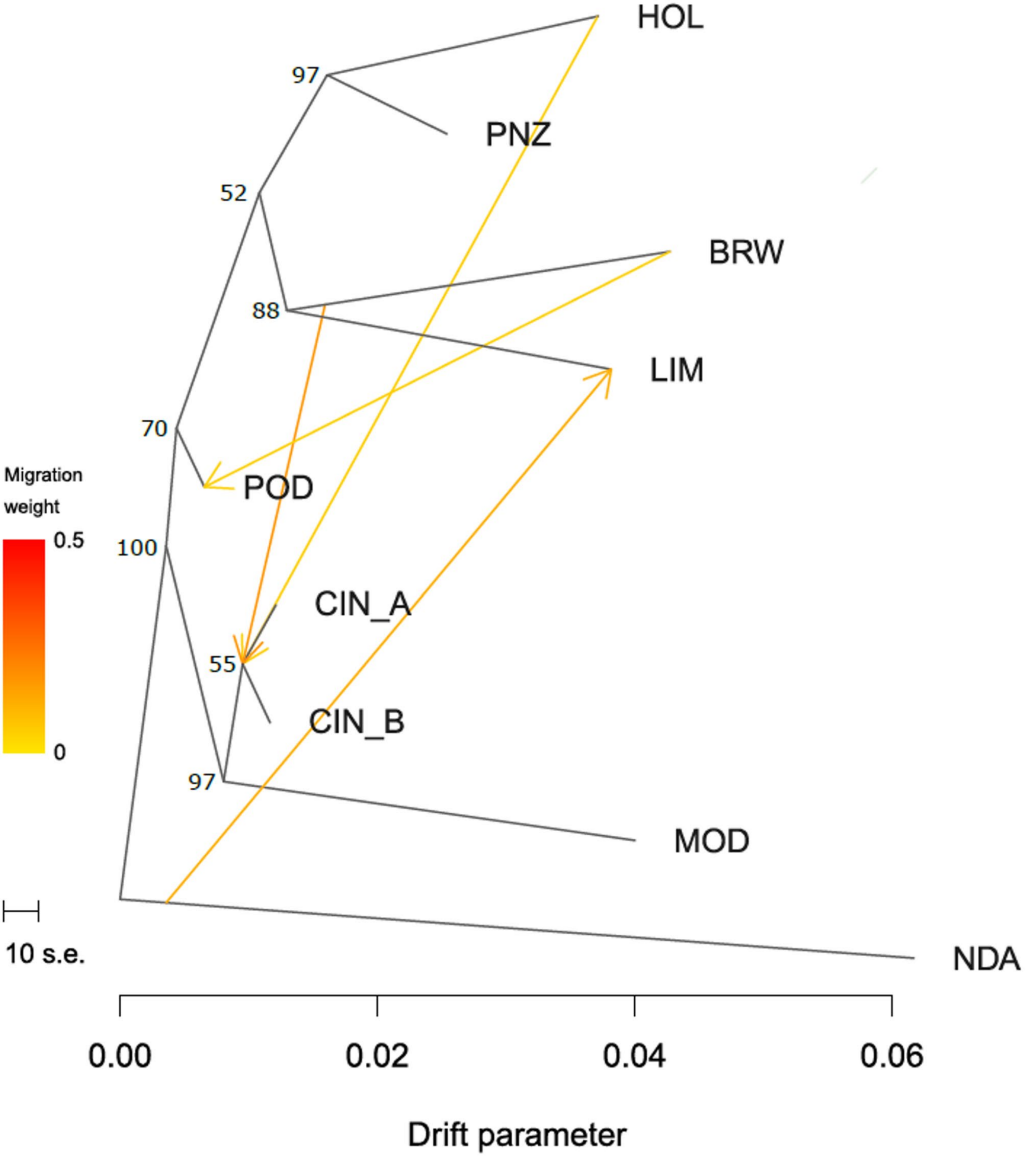
Phylogenetic networks inferred by Treemix showing the relationship among populations. The migration edges, with arrows pointing in the direction toward the recipient group, are coloured according to the ancestry percentage received from the donor. Bootstrap values are shown next to each node

The assessment of population stratification performed with Admixture aligned with the Treemix results. OptM identified four as the optimal number of gene flow events (Fig. 2). The first two migration events identified gene flow sourcing from HOL and BRW to the node from which Cinisara stems, the third identified a gene flow vector sourcing from the tip of BRW to POD, and the fourth sourced from the base of NDA to the tip of LIM.

Haplotype sharing analysis recorded the highest median total length of shared IBD segments (59 Mb) between HOL and PNZ, whereas much lower values were observed between CIN_A and CIN_B (26 Mb) (Additional File 4). HOL and MOD were the breeds showing the highest haplotype sharing with both CIN_A (10 and 9 Mb, respectively) and CIN_B (9.4 and 9 Mb, respectively). Interestingly, haplotype segments sourcing from BRW were higher in CIN_B than CIN_A (7.6 and 5.4 Mb, respectively), whereas CIN_A exhibited slightly higher sharing with PNZ (5.7 Mb). Similarly to what was observed for F_ROH_, the distribution of the length of shared IBD segments with BRW and, especially, HOL was more variable among CIN_B than CIN_A subjects. Notably, the *f3* statistics did not detect any gene flow signal surpassing the significance threshold.

### Local ancestry inference

In both Cinisara populations, we observed higher local introgression from HOL compared to BRW. The mean local ancestry proportions for HOL and BRW were 0.127 and 0.054, respectively, in CIN_A and 0.112 and 0.060 in CIN_B (Additional File 5 and 6; Table S1 and S2). However, CIN_B exhibited greater heterogeneity in mean introgression levels from both HOL and BRW compared to CIN_A (Additional file 6 - Table S3).

**Fig. 3:**
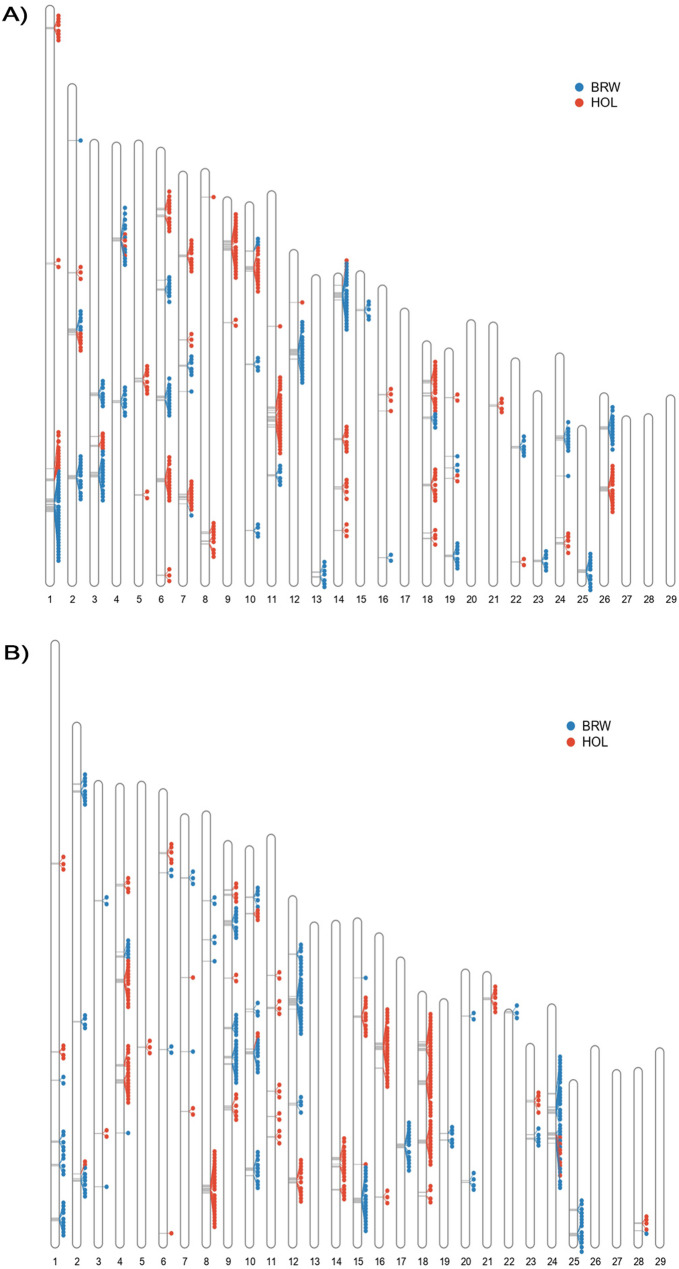
Ideogram illustrating the distribution of highly introgressed (99^th^ percentile) regions in the bovine autosomes of the CIN_A (**A**) and CIN_B (**B**) populations. Potential candidate regions of Holstein (red dots) or Brown Swiss (blue dots) origin are plotted

Among the SNPs classified as highly introgressed (99^th^ percentiles) in CIN_A, 258 and 257 were found using HOL and BRW as reference, intercepting 186 and 131 genes, and 1,584 and 1,772 QTLs, respectively (Fig. 3a; Additional file 6 and Table S4-S8). Similarly, in CIN_B, we counted 256 and 254 highly introgressed SNPs associated with the HOL and BRW references, intercepting 218 and 184 genes and 547 and 437 QTLs for HOL and BRW, respectively (Fig. 3b; Additional file 6 and Table S9-S13). The identified QTLs in CIN_A were predominantly located on chromosomes 6, 14, and 26, whereas those in CIN_B were primarily found on chromosomes 11, 14 and 18 (Additional file 7). A total of 43 and 21 genes were shared between CIN_A and CIN_B among the highly introgressed SNPs originating from HOL and BRW, respectively (Additional file 6 and Tables S14 and S15).

Overall, the number of genes in common between the two groups of Cinisara was higher for HOL than BRW and many of the genes found in both groups or a single Cinisara group were associated with relevant traits for cattle breeding. Specifically, among the genes found in HOL derived regions identified in both Cinisara groups, *CDC42BPA* and *MYLK3* are known to regulate milk fat percentage [53] and milk production traits [54]. Other genes identified in HOL introgressed regions in both Cinisara groups were associated with growth traits, with *ANKRD11* and *MYLK3* involved with muscle development and structure in several cattle breeds [55, 56].

Genes found to be highly introgressed from BRW in both Cinisara groups were also associated with reproduction, such as *SMAD6*, a major gene involved in the bovine ovulation rate and production traits [57]. Several genes are related with production traits, such as: *ASXL3*, highly expressed in the mammary gland and associated with lipid metabolism and milk quality [58], *CPNE4*, related with dry days and growth [59], and *STARD13* [60]. Among those genes found to be highly introgressed from HOL in CIN_A, *FAIM* is associated with muscle and fat traits in cattle [61], along with *HADH*, linked to intramuscular fat deposition [62], and *CCDC158*, associated with carcass quality traits [63]. Importantly, we identified *DNAJC13* and *LEF1*, two genes associated with thermotolerance. *DNAJC13*, derived from BRW introgression in CIN_A, encodes a member of the Dnaj protein family, which acts as co-chaperone to heat-shock proteins and has been implicated in heat resistance in cattle [64]. *LEF1*, located in HOL-introgressed regions in both CIN_A and CIN_B, is a transcriptional factor of the Wnt/Lrp5/beta-catenin signalling cascade regulating bone remodelling and regeneration, and mammary gland development, but is also associated to immune response, thermotolerance and UV protection in cattle [65–68].

## Discussion

The Cinisara breed is a local dual-purpose Sicilian population with a complex history of management and registration, which has influenced its relationships with other Italian and cosmopolitan breeds. This complexity is reflected in the results of the present study. Both PCA and Admixture analyses are suggestive of a Podolian lineage for the Cinisara breed, confirming previous observations linking Cinisara to Podolian breeds from the Balkans, such as the Serbian Podolsko, Croatian Podolian, and Hungarian Grey [19]. This is further aligned with historical accounts of cattle from southern Italy being used to replenish Sicilian herds around 1860 [20]. The relationship between Cinisara and Modicana — two local Sicilian breeds — is suggested by both PCA and Neighbour-Net analyses, confirming findings by previous studies [29]. We hypothesize that this relationship stems from the historical inclusion of Cinisara in the Modicana herdbook prior to its recognition as a distinct breed, potentially facilitating gene flow between the two. Moreover, the investigation of Cinisara genomic make-up also confirmed the alleged crossbreeding with cosmopolitan breeds aimed at improving production performance. Generally, we observed an unexpected lack of correlation between F_ROH_ and N_e_. This is probably related to the different genetic variability captured by the two analyses and the intensity and durations of demographic events, which produce distinct signatures and lead to heterogeneous ROH patterns that may not align with the genome-wide signals captured by N_e_.

To evaluate the impact of these breeding practices on the breed’s conservation, we assessed the genetic health of the current Cinisara population and compared it with a cohort from approximately 20 years ago. Despite being a geographically restricted, small-sized breed currently lacking a well-structured monitoring plan, both Cinisara populations exhibited genetic diversity indices comparable to those of larger, better-managed populations. However, CIN_B displayed lower heterozygosity, effective population size, and overall genetic diversity, coupled with higher inbreeding levels compared to CIN_A, while also exhibiting greater variability in these parameters across individuals. Additionally, although mean introgression from HOL and BRW appeared higher in CIN_A, as supported by admixture analyses, haplotype sharing patterns, and local ancestry proportion estimates, CIN_B confirmed higher heterogeneity. In the absence of official guidelines for genetic management programs in this population, the observed findings — higher introgression and greater diversity in CIN_A, but higher variability in CIN_B — can be attributed to three main factors: differences in the populations and lineages sampled, improved management of Cinisara in recent decades (despite the lack of formal genetic programs), and the introduction of new alleles in CIN_A through ongoing crossbreeding with cosmopolitan breeds. These factors may also act in combination, with CIN_B representing an earlier stage of the crossbreeding process, where some individuals underwent introgression, while others, characterised by higher inbreeding and lower genetic sharing with HOL and BRW, did not. Conversely, CIN_A appears to represent a more homogeneously introgressed population. Moreover, HOL emerged as the primary source of introgression in both Cinisara populations, with indications of a relatively greater contribution of BRW introgression in CIN_B compared to CIN_A. This result, especially evident in the haplotype sharing results, might reflect a decline in crossbreeding with BRW or a preference for HOL introgression in recent years. A possible explanation might be the change in the availability of BRW breeding bulls though times, or a directional choice towards HOL higher productivity. However, providing a definitive interpretation is challenging, as more data is necessary to accurately reconstruct the time and extent of the introgression patterns.

The evidence of introgression from cosmopolitan breeds into a local population is significant, as it highlights the potential consequences of population mismanagement and the absence of structured genetic programs. Over time, this could lead to the erosion of unique and adaptive traits, such as environmental adaptation. To address this, we examined the highly introgressed SNPs and observed that those identified in both Cinisara populations were associated with traits relevant to cattle breeding, such as milk yield, and protein and fat content. These findings might align with farmers’ efforts to enhance milk profitability. Among the genes found in either HOL (e.g., *CDC42BPA* and *MYLK3)* or BRW (*ASXL3*, *CPNE4*, *STARD13*) derived regions, identified in both Cinisara groups, several are associated with milk production traits. Importantly, although having a lower milk yield, Cinisara milk is characterized by higher protein and coagulation properties than HOL and BRW [23, 24, 69, 70]. Therefore, it is possible that while improving milk quantity, the identified cosmopolitan breed introgression might collaterally reduce Cinisara milk quality.

Some genes identified in HOL introgressed regions in both Cinisara groups (*ANKRD11*,* MYLK3*) or uniquely in CIN_A (*FAIM*,* HADH*,* CCDC158*) are associated with growth-related traits. Considering the dairy-oriented specialization of the Holstein breed, it can be hypothesized that these introgressions may alter the traditional dual-purpose characteristic of Cinisara or potentially compromise its overall performance. In light of our results, future studies could explore potential changes in milk quality and meat production performance in Cinisara over the last decades to better understand the possible effects of introgression on the breed’s productive traits. Additionally, among the genes identified as introgressed by HOL and BRW, two (*LEF1* and *DNAJC13*, respectively) are described as associated with heat resistance in cattle [64, 65, 67]. As highly managed breeds, the introgression of gene variants from HOL and BRW may adversely affect the adaptive capacity of Cinisara cattle by displacing evolved variants that are better suited to the hot and arid Sicilian climate.

## Conclusions

Here we demonstrated that while introgression with Holstein and Brown Swiss has likely contributed to increased dairy production traits in Cinisara, it may also have undermined the breed’s unique genetic diversity and adaptive characteristics specific to the harsh Sicilian environment, a critical vulnerability under a rapidly changing climate. These results are particularly worrying considering the small census and effective population size of Cinisara, coupled with a restricted range. However, it is crucial to recognize that the success of any conservation strategy lies in the balance with the economic realities faced by local farmers, whose livelihood often depends on the economic viability of these animals. Therefore, future research and management efforts must prioritize a balanced approach integrating scientific principles with practical solutions for farmers, such as developing breeding strategies that optimize both production and genetic conservation. As shown in this work, genomics can play an instrumental role both in monitoring the population and supporting educated breeding strategies, e.g., by revealing unduly practices and selecting those animals less impacted by cosmopolitan breed introgression for breeding. Ultimately, the long-term sustainability of the Cinisara population relies on the collaborative effort among scientists, farmers, and local policymakers. By fostering a framework that acknowledges both the scientific and socioeconomic dimensions of breed conservation, we can ensure the preservation of this valuable genetic resource for future generations.

## Supplementary Information

Additional file 1. Distribution of inbreeding valuesfor all groups.

Additional file 2. Scatter Plot of the first two principal componentsof the principal component analysisperformed excluding the N’Damabreed.

Additional file 3. Admixture plot from K 2 to 8 considering all breeds studied.

Additional file 4. IBD haplotype sharing of all the analysed breeds.

Additional file 5. Local ancestry inferenceconsidering three reference populations: POD as a representative of the ancestral Podolian background, and HOL and BRW that were identified by the former gene flow analyses as candidate sources of introgression. Each Cinisara populationwas individually tested as target population.

Additional file 6. Table S1-List of introgressed SNPs identified in the Cinisara cattle breed. For each SNP, the following information is given: the bovine chromosome, the position of the SNP, the flanking region, the proportion of introgression given by each reference population and the target population. Table S2-List of introgressed SNPs identified in the Cinisara cattle breed. For each SNP, the following information is given: the bovine chromosome, the position of the SNP, the flanking region, the proportion of introgression given by each reference population and the target population. Table S3 - Mean FROH, average individual introgression from HOL and BRW as calculated with lai analysis, and mean total length of IBD sharing with HOL and BRW of CIN_A and CIN_B individuals. Table S4 - List of introgressed SNPs identified in the 99th percentile in the Cinisaracattle breed. For each SNP, the following information is given: the bovine chromosome, the position of the SNP, the flanking region, the proportion of introgression given by each reference population and target population. Table S5 - List of genes located on highly introgressed SNP of Cinisara Ausing Holstein as reference population. The bovine chromosomeon which the SNP and genes are located, the positionsfor SNP start and SNP end, the positionsfor gene start and gene end and the gene names are given. Table S6 - List of genes located on the highly introgressed SNP of Cinisara Ausing Brown Swiss as reference population. The bovine chromosomeon which the SNP and genes are located, the positionsfor SNP start and SNP end, the positionsfor gene start and gene end and the gene names are given. Table S7 - List of the highly introgressed SNP identified in CIN_A using Holstein as reference population located on the quantitative traits loci. The chromosome and start and end position of SNPs, the chromosome, start and end position and the description of QTL are given. Table S8 - List of the highly introgressed SNP identified in CIN_A using Brown Swiss as reference population located on the quantitative traits loci. The chromosome and start and end position of SNPs, the chromosome, start and end position and the description of QTL are given. Table S9 - List of introgressed SNPs identified in the 99th percentile in the Cinisaracattle breed. For each SNP, the following information is given: the bovine chromosome, the position of the SNP, the flanking region, the proportion of introgression given by each reference population and target population. Table S10 - List of genes located on the candidate regions of Cinisara Ausing Holstein as reference population. The bovine chromosomeon which the SNP and genes are located, the positionsfor SNP start and SNP end, the positionsfor gene start and gene end and the gene names are given. Table S11 - List of genes located on the candidate regions of Cinisara Ausing Brown Swiss as reference population. The bovine chromosomeon which the SNP and genes are located, the positionsfor SNP start and SNP end, the positionsfor gene start and gene end and the gene names are given. Table S12 - List of the highly introgressed SNP identified in CIN_B using Holstein as reference population located on the quantitative traits loci. The chromosome and start and end position of SNPs, the chromosome, start and end position and the description of QTL are given. Table S13 - List of the highly introgressed SNP identified in CIN_B using Brown Swiss as reference population located on the quantitative traits loci. The chromosome and start and end position of SNPs, the chromosome, start and end position and the description of QTL are given. Table S14 - List of genes in common between CIN_A and CIN_B in the highly introgressive SNPs from HOL. The bovine chromosomeon which the SNP and genes are located, the positionsfor SNP start and SNP end, the positionsfor gene start and gene end and the gene names are given. Table S15 - List of genes in common between CIN_A and CIN_B in the highly introgressive SNPs from BRW. The bovine chromosomeon which the SNP and genes are located, the positionsfor SNP start and SNP end, the positionsfor gene start and gene end and the gene names are given.

Additional file 7. Sum of QTLs identified in highly introgressed regions in both populations by HOL and BRW.

Additional file 8. Genotype data of Cinisara, Limousine and Modicana.

## Data Availability

The genotype data generated for this study are available as Additional file 8.

## References

[1] Hoffmann I. Adaptation to climate change– exploring the potential of locally adapted breeds. Animal. 2013;7:346–62. 23739476 10.1017/S1751731113000815

[2] Barbato M, Hailer F, Upadhyay M, Del Corvo M, Colli L, Negrini R, et al. Adaptive introgression from indicine cattle into white cattle breeds from central Italy. Sci Rep. 2020;10:1279. 31992729 10.1038/s41598-020-57880-4 PMC6987186

[3] Bruford MW, Ginja C, Hoffmann I, Joost S, Orozco-terWengel P, Alberto FJ et al. Prospects and challenges for the conservation of farm animal genomic resources, 2015–2025. Front Genet. 2015;6. 10.3389/fgene.2015.00314. 26539210 10.3389/fgene.2015.00314 PMC4612686

[4] Somenzi E, Partel E, Barbato M, Chero Osorio AM, Colli L, Franceschi N, et al. Genetic legacy and adaptive signatures: investigating the history, diversity, and selection signatures in Rendena cattle resilient to eighteenth century Rinderpest epidemics. Genet Sel Evol. 2024;56:32. 38698323 10.1186/s12711-024-00900-y PMC11064358

[5] Di Trana A, Sepe L, Di Gregorio P, Di Napoli MA, Giorgio D, Caputo AR, et al. The role of local sheep and goat breeds and their products as a tool for sustainability and safeguard of the mediterranean environment. In: Vastola A, editor. The sustainability of Agro-Food and natural resource systems in the mediterranean basin. Cham: Springer International Publishing; 2015. pp. 77–112.

[6] Gandini GC, Villa E. Analysis of the cultural value of local livestock breeds: a methodology. J Anim Breed Genet. 2003;120:1–11.

[7] Sponenberg DP, Beranger J, Martin AM, Couch CR. Conservation of rare and local breeds of livestock. Rev Sci Tech OIE. 2018;37:259–67. 10.20506/rst.37.1.2756 30209434

[8] Biscarini F, Nicolazzi EL, Stella A, Boettcher PJ, Gandini G. Challenges and opportunities in genetic improvement of local livestock breeds. Front Genet. 2015;6. 10.3389/fgene.2015.00033. 25763010 10.3389/fgene.2015.00033 PMC4340267

[9] Joy A, Dunshea FR, Leury BJ, Clarke IJ, DiGiacomo K, Chauhan SS. Resilience of small ruminants to climate change and increased environmental temperature: A review. Animals. 2020;10:867. 32429527 10.3390/ani10050867 PMC7278399

[10] O’Donnell DR, Parigi A, Fish JA, Dworkin I, Wagner AP. The roles of standing genetic variation and evolutionary history in determining the evolvability of Anti-Predator strategies. PLoS ONE. 2014;9:e100163. 24955847 10.1371/journal.pone.0100163 PMC4067307

[11] Pulina G, Milán MJ, Lavín MP, Theodoridis A, Morin E, Capote J, et al. Invited review: current production trends, farm structures, and economics of the dairy sheep and goat sectors. J Dairy Sci. 2018;101:6715–29. 29859690 10.3168/jds.2017-14015

[12] Ilea RC. Intensive livestock farming: global trends, increased environmental concerns, and ethical solutions. J Agric Environ Ethics. 2009;22:153–67.

[13] Molnár M. Transforming intensive animal production: challenges and opportunities for farm animal welfare in the European union. Animals. 2022;12:2086. 36009676 10.3390/ani12162086 PMC9404898

[14] Professor of Applied Philosophy, University W, University F, Netherlands, Korthals M. Is Intensive Farming Ethically Acceptable? AS. 2018;2:15–29.

[15] Burhans WS, Rossiter Burhans CA, Baumgard LH. Invited review: lethal heat stress: the putative pathophysiology of a deadly disorder in dairy cattle. J Dairy Sci. 2022;105:3716–35. 35248387 10.3168/jds.2021-21080

[16] Kadzere CT, Murphy MR, Silanikove N, Maltz E. Heat stress in lactating dairy cows: a review. Livest Prod Sci. 2002;77:59–91.

[17] Habimana V, Nguluma AS, Nziku ZC, Ekine-Dzivenu CC, Morota G, Mrode R, et al. Heat stress effects on milk yield traits and metabolites and mitigation strategies for dairy cattle breeds reared in tropical and sub-tropical countries. Front Vet Sci. 2023;10:1121499. 37483284 10.3389/fvets.2023.1121499 PMC10361820

[18] Hoffmann I. Climate change and the characterization, breeding and conservation of animal genetic resources. Anim Genet. 2010;41:32–46. 20500754 10.1111/j.1365-2052.2010.02043.x

[19] Senczuk G, Mastrangelo S, Ajmone-Marsan P, Becskei Z, Colangelo P, Colli L, et al. On the origin and diversification of Podolian cattle breeds: testing scenarios of European colonization using genome-wide SNP data. Genet Sel Evol. 2021;53:48. 34078254 10.1186/s12711-021-00639-w PMC8173809

[20] Chicoli N. Riproduzione, allevamento e miglioramento degli animali domestici in Sicilia. 1870.

[21] Bonanno A, Tornambè G, Bellina V, De Pasquale C, Mazza F, Maniaci G, et al. Effect of farming system and cheesemaking technology on the physicochemical characteristics, fatty acid profile, and sensory properties of Caciocavallo Palermitano cheese. J Dairy Sci. 2013;96:710–24. 23127907 10.3168/jds.2012-5973

[22] Caracappa S, Cannizzo FT, Caracappa G, Sacchi P. Vi raccontiamo Le razze: La Bovina Cinisara o Vacca Nera Siciliana. 2023. https://www.ruminantia.it/vi-raccontiamo-le-razze-la-bovina-cinisara-o-vacca-nera-siciliana/.

[23] Di Gregorio P, Di Grigoli A, Di Trana A, Alabiso M, Maniaci G, Rando A, et al. Effects of different genotypes at the CSN3 and LGB loci on milk and cheese-making characteristics of the bovine Cinisara breed. Int Dairy J. 2017;71:1–5.

[24] Altomonte I, Salari F, Neglia A, Martini M. Milk yield and quality characteristics of Cinisara and modicana cows reared on a farm in the Province of Palermo (Sicily-Italy). 2016.

[25] Alabiso M, Maniaci G, Giosuè C, Di Grigoli A, Bonanno A. Fatty acid composition of Salami made by meat from different commercial categories of Indigenous dairy cattle. Animals. 2021;11:1060. 33918052 10.3390/ani11041060 PMC8069036

[26] Alabiso M, Maniaci G, Giosuè C, Gaglio R, Francesca N, Di Grigoli A, et al. Effect of muscle type and animal category on fatty acid composition of Bresaola made from meat of Cinisara cattle: preliminary investigation. CyTA - J Food. 2020;18:734–41.

[27] Gaglio R, Francesca N, Maniaci G, Corona O, Alfonzo A, Giosuè C, et al. Valorization of Indigenous dairy cattle breed through Salami production. Meat Sci. 2016;114:58–68. 26735574 10.1016/j.meatsci.2015.12.014

[28] Liotta L, Chiofalo V, D’Alessandro E, Vasi S, Ferrante A, Chiofalo B. Slaughtering traits and meat quality of Cinisara cattle native Italian breed. Italian J Anim Sci. 2011;10:e28.

[29] Mastrangelo S, Ciani E, Ajmone Marsan P, Bagnato A, Battaglini L, Bozzi R, et al. Conservation status and historical relatedness of Italian cattle breeds. Genet Sel Evol. 2018;50:35. 29940848 10.1186/s12711-018-0406-x PMC6019226

[30] Sempéré G, Moazami-Goudarzi K, Eggen A, Laloë D, Gautier M, Flori L. WIDDE: a Web-Interfaced next generation database for genetic diversity exploration, with a first application in cattle. BMC Genomics. 2015;16:940. 26573482 10.1186/s12864-015-2181-1 PMC4647285

[31] Chang CC, Chow CC, Tellier LC, Vattikuti S, Purcell SM, Lee JJ. Second-generation PLINK: rising to the challenge of larger and richer datasets. GigaSci. 2015;4:7. 10.1186/s13742-015-0047-8 PMC4342193 25722852

[32] Manichaikul A, Mychaleckyj JC, Rich SS, Daly K, Sale M, Chen W-M. Robust relationship inference in genome-wide association studies. Bioinformatics. 2010;26:2867–73. 20926424 10.1093/bioinformatics/btq559 PMC3025716

[33] Meyermans R, Gorssen W, Buys N, Janssens S. How to study runs of homozygosity using PLINK? A guide for analyzing medium density SNP data in livestock and pet species. BMC Genomics. 2020;21:94. 31996125 10.1186/s12864-020-6463-x PMC6990544

[34] Ferenčaković M, Sölkner J, Curik I. Estimating autozygosity from high-throughput information: effects of SNP density and genotyping errors. Genet Sel Evol. 2013;45:42. 24168655 10.1186/1297-9686-45-42 PMC4176748

[35] Purfield DC, Berry DP, McParland S, Bradley DG. Runs of homozygosity and population history in cattle. BMC Genet. 2012;13:70. 22888858 10.1186/1471-2156-13-70 PMC3502433

[36] Browning BL, Tian X, Zhou Y, Browning SR. Fast two-stage phasing of large-scale sequence data. Am J Hum Genet. 2021;108:1880–90. 34478634 10.1016/j.ajhg.2021.08.005 PMC8551421

[37] McQuillan R, Leutenegger A-L, Abdel-Rahman R, Franklin CS, Pericic M, Barac-Lauc L, et al. Runs of homozygosity in European populations. Am J Hum Genet. 2008;83:359–72. 18760389 10.1016/j.ajhg.2008.08.007 PMC2556426

[38] Barbato M, Orozco-terWengel P, Tapio M, Bruford MW. SNeP: a tool to estimate trends in recent effective population size trajectories using genome-wide SNP data. Front Genet. 2015;6. 10.3389/fgene.2015.00109. 25852748 10.3389/fgene.2015.00109 PMC4367434

[39] Pitt D, Bruford MW, Barbato M, Orozco-terWengel P, Martínez R, Sevane N. Demography and rapid local adaptation shape Creole cattle genome diversity in the tropics. Evol Appl. 2019;12:105–22. 30622639 10.1111/eva.12641 PMC6304683

[40] Huson DH, Bryant D. Application of phylogenetic networks in evolutionary studies. Mol Biol Evol. 2006;23:254–67. 16221896 10.1093/molbev/msj030

[41] Alexander DH, Novembre J, Lange K. Fast model-based Estimation of ancestry in unrelated individuals. Genome Res. 2009;19:1655–64. 19648217 10.1101/gr.094052.109 PMC2752134

[42] Kopelman NM, Mayzel J, Jakobsson M, Rosenberg NA, Mayrose I. Clumpak: a program for identifying clustering modes and packaging population structure inferences across *K*. Mol Ecol Resour. 2015;15:1179–91. 25684545 10.1111/1755-0998.12387 PMC4534335

[43] Pickrell JK, Pritchard JK. Inference of population splits and mixtures from Genome-Wide allele frequency data. PLoS Genet. 2012;8:e1002967. 23166502 10.1371/journal.pgen.1002967 PMC3499260

[44] Fitak RR. *OptM*: estimating the optimal number of migration edges on population trees using *Treemix*. Biology Methods Protocols. 2021;6:bpab017. 34595352 10.1093/biomethods/bpab017 PMC8476930

[45] Cummings MP. PHYLIP (PHYLogeny Inference Package). In: Hancock JM, Zvelebil MJ, editors. Dictionary of Bioinformatics and Computational Biology. 1st edition. Wiley; 2004. 10.1002/9780471650126.dob0534.pub2.

[46] Browning BL, Browning SR. Improving the accuracy and efficiency of Identity-by-Descent detection in population data. Genetics. 2013;194:459–71. 23535385 10.1534/genetics.113.150029 PMC3664855

[47] Gu Z, Gu L, Eils R, Schlesner M, Brors B. Circlize implements and enhances circular visualization in R. Bioinformatics. 2014;30:2811–2. 24930139 10.1093/bioinformatics/btu393

[48] Guan Y. Detecting structure of haplotypes and local ancestry. Genetics. 2014;196:625–42. 24388880 10.1534/genetics.113.160697 PMC3948796

[49] Hao Z, Lv D, Ge Y, Shi J, Weijers D, Yu G, et al. *RIdeogram*: drawing SVG graphics to visualize and map genome-wide data on the idiograms. PeerJ Comput Sci. 2020;6:e251. 33816903 10.7717/peerj-cs.251 PMC7924719

[50] Quinlan AR, Hall IM. BEDTools: a flexible suite of utilities for comparing genomic features. Bioinformatics. 2010;26:841–2. 20110278 10.1093/bioinformatics/btq033 PMC2832824

[51] Hu Z-L, Park CA, Reecy JM. Bringing the animal QTLdb and CorrDB into the future: meeting new challenges and providing updated services. Nucleic Acids Res. 2022;50:D956–61. 34850103 10.1093/nar/gkab1116 PMC8728226

[52] Mastrangelo S, Saura M, Tolone M, Salces-Ortiz J, Di Gerlando R, Bertolini F, et al. The genome-wide structure of two economically important Indigenous Sicilian cattle breeds1. J Anim Sci. 2014;92:4833–42. 25253807 10.2527/jas.2014-7898

[53] Jiang J, Ma L, Prakapenka D, VanRaden PM, Cole JB, Da Y. A Large-Scale Genome-Wide association study in U.S. Holstein cattle. Front Genet. 2019;10:412. 31139206 10.3389/fgene.2019.00412 PMC6527781

[54] Saravanan KA, Panigrahi M, Kumar H, Parida S, Bhushan B, Gaur GK, et al. Genomic scans for selection signatures revealed candidate genes for adaptation and production traits in a variety of cattle breeds. Genomics. 2021;113:955–63. 33610795 10.1016/j.ygeno.2021.02.009

[55] He Y, Huang Y, Wang S, Zhang L, Gao H, Zhao Y, et al. Hereditary basis of coat color and excellent feed conversion rate of red Angus cattle by Next-Generation sequencing data. Animals. 2022;12:1509. 35739846 10.3390/ani12121509 PMC9219544

[56] Liu S, Gao Y, Canela-Xandri O, Wang S, Yu Y, Cai W, et al. A multi-tissue atlas of regulatory variants in cattle. Nat Genet. 2022;54:1438–47. 35953587 10.1038/s41588-022-01153-5 PMC7613894

[57] Kirkpatrick BW, Morris CA. A major gene for bovine ovulation rate. PLoS ONE. 2015;10:e0129025. 26046917 10.1371/journal.pone.0129025 PMC4457852

[58] Sanchez M-P, Ramayo-Caldas Y, Wolf V, Laithier C, El Jabri M, Michenet A, et al. Sequence-based GWAS, network and pathway analyses reveal genes co-associated with milk cheese-making properties and milk composition in Montbéliarde cows. Genet Sel Evol. 2019;51:34. 31262251 10.1186/s12711-019-0473-7 PMC6604208

[59] Ahmad SF, Singh A, Gangwar M, Kumar S, Dutt T, Kumar A. Haplotype-based association study of production and reproduction traits in multigenerational Vrindavani population. Gene. 2023;867:147365. 36918047 10.1016/j.gene.2023.147365

[60] Ibeagha-Awemu EM, Ibeagha AE, Zhao X. The influence of different anticoagulants and sample Preparation methods on measurement of mCD14 on bovine monocytes and polymorphonuclear neutrophil leukocytes. BMC Res Notes. 2012;5:93. 22333045 10.1186/1756-0500-5-93 PMC3312831

[61] Wang L, Liu X, Wang H, He H, Li Z, Chen L. Expression analysis, single nucleotide polymorphisms and combined genotypes in candidate genes and their associations with growth and carcass traits in Qinchuan cattle. Mol Biol Rep. 2013;40:2335–46. 23196708 10.1007/s11033-012-2315-3

[62] Xiong L, Pei J, Chu M, Wu X, Kalwar Q, Yan P, et al. Fat deposition in the muscle of female and male Yak and the correlation of Yak meat quality with fat. Animals. 2021;11:2142. 34359275 10.3390/ani11072142 PMC8300776

[63] Lee J-Y, Lee J-H, Yeo J-S, Kim J-J. A SNP harvester analysis to better detect SNPs of CCDC158 gene that are associated with carcass quality traits in Hanwoo. Asian Australas J Anim Sci. 2013;26:766–71. 25049848 10.5713/ajas.2012.12715 PMC4093242

[64] Guan X, Zhao S, Xiang W, Jin H, Chen N, Lei C, et al. Genetic diversity and selective signature in Dabieshan cattle revealed by Whole-Genome resequencing. Biology. 2022;11:1327. 36138806 10.3390/biology11091327 PMC9495734

[65] Gao Y, Gautier M, Ding X, Zhang H, Wang Y, Wang X, et al. Species composition and environmental adaptation of Indigenous Chinese cattle. Sci Rep. 2017;7:16196. 29170422 10.1038/s41598-017-16438-7 PMC5700937

[66] Xing S, Gai K, Li X, Shao P, Zeng Z, Zhao X, et al. Tcf1 and Lef1 are required for the immunosuppressive function of regulatory T cells. J Exp Med. 2019;216:847–66. 30837262 10.1084/jem.20182010 PMC6446865

[67] Lyu Y, Guan X, Xu X, Wang P, Li Q, Panigrahi M, et al. A whole genome scan reveals distinct features of selection in Zhaotong cattle of Yunnan Province. Anim Genet. 2023;54:731–42. 37796667 10.1111/age.13363

[68] Sahu S, Sahoo S, Sullivan T, O’Sullivan TN, Turan S, Albaugh ME, et al. Spatiotemporal modulation of growth factors directs the generation of multilineage mouse embryonic stem cell-derived mammary organoids. Dev Cell. 2024;59:175–e1868. 38159568 10.1016/j.devcel.2023.12.003 PMC10872289

[69] Franceschi P, Malacarne M, Formaggioni P, Faccia M, Summer A. Quantification of the effect of the cattle breed on milk cheese yield: comparison between Italian brown Swiss and Italian Friesian. Animals. 2020;10:1331. 32752195 10.3390/ani10081331 PMC7459824

[70] Malacarne M, Faccia M, Rossoni A, Santus E, Formaggioni P, Summer A, et al. New insights in cheese yield capacity of the milk of Italian brown and Italian Friesian cattle in the production of High-Moisture Mozzarela. Food Technol Biotechnol (Online). 2020;58:91–7. 10.17113/ftb.58.01.20.6386 PMC7365334 32684793

