# Supplementary figures and images for: Uncovering the architecture of production-driven introgression in Cinisara cattle breed
Source: BMC Genom Data. 2025 Jul 11;26:47. doi: 10.1186/s12863-025-01337-y (PMC12247468; doi:10.1186/s12863-025-01337-y)

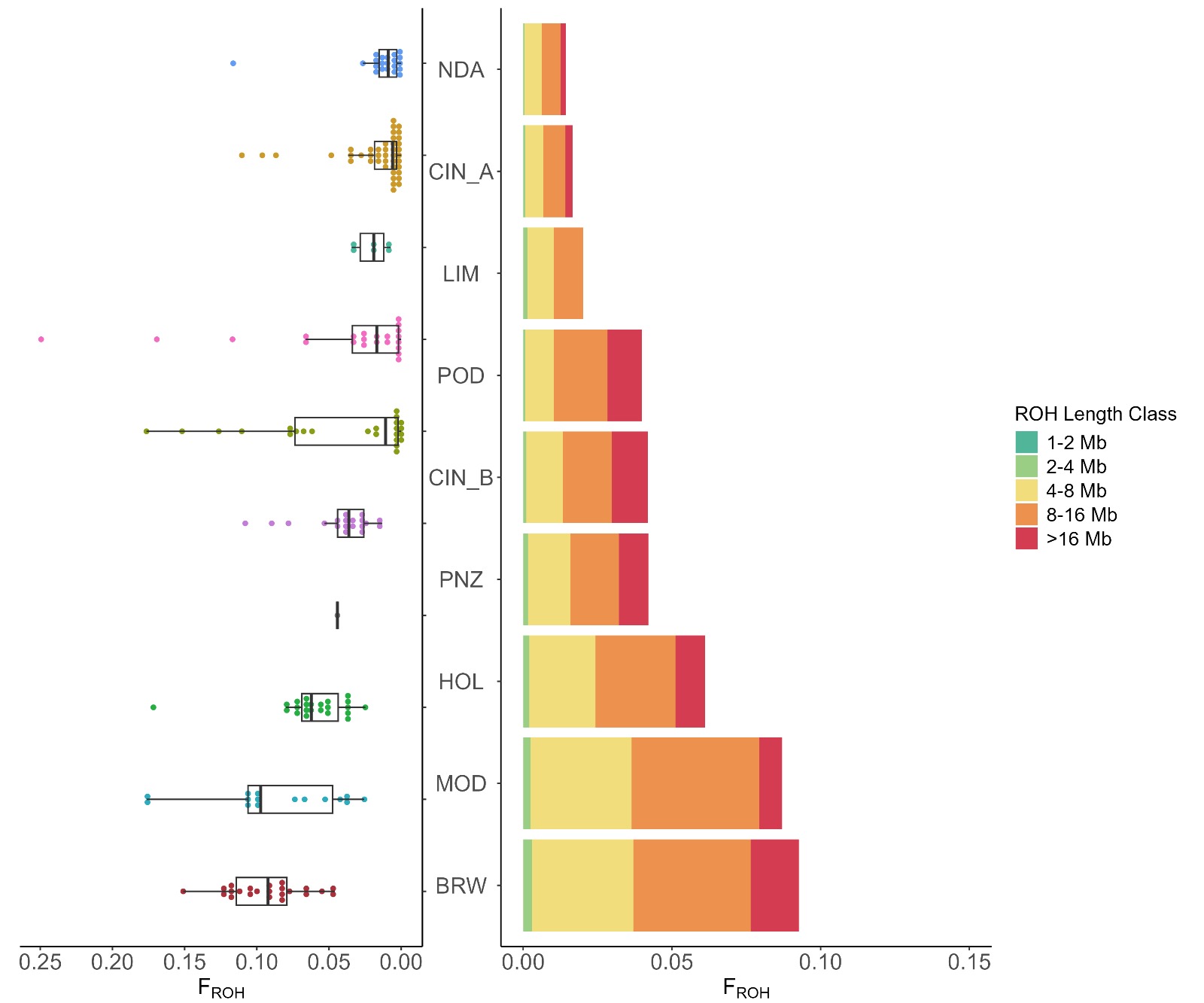

Supplement: Supplementary file 1 — Additional file 1. Distribution of inbreeding valuesfor all groups. [file 12863_2025_1337_MOESM1_ESM.jpg]

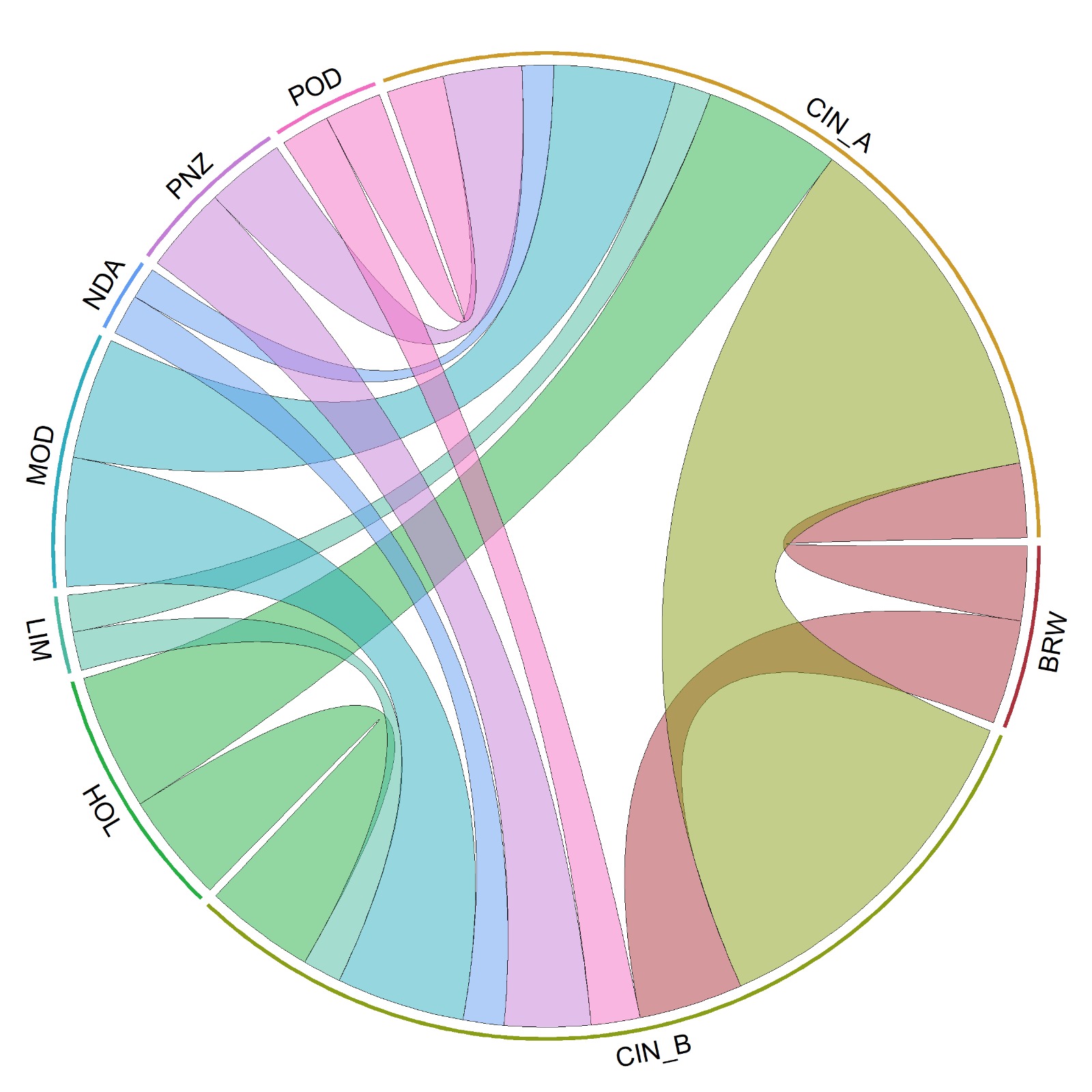

Supplement: Supplementary file 4 — Additional file 4. IBD haplotype sharing of all the analysed breeds. [file 12863_2025_1337_MOESM4_ESM.jpg]

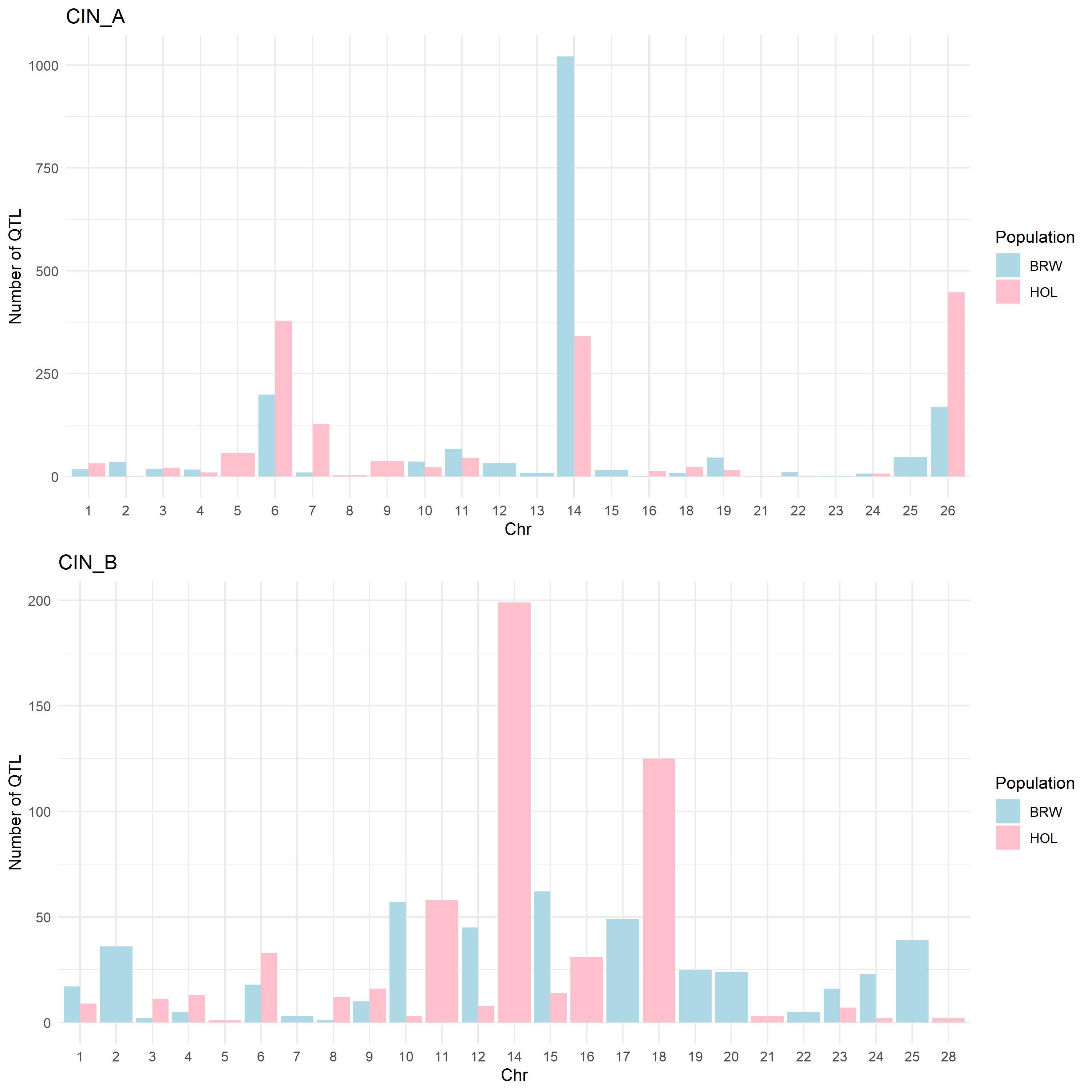

Supplement: Supplementary file 7 — Additional file 7. Sum of QTLs identified in highly introgressed regions in both populations by HOL and BRW. [file 12863_2025_1337_MOESM7_ESM.png]
