# Supplementary material for: Uncovering the architecture of production-driven introgression in Cinisara cattle breed
Source: BMC Genom Data. 2025 Jul 11;26:47. doi: 10.1186/s12863-025-01337-y (PMC12247468; doi:10.1186/s12863-025-01337-y)
Supplement: Supplementary file 3 — Additional file 3. Admixture plot from K 2 to 8 considering all breeds studied. [file 12863_2025_1337_MOESM3_ESM.pdf]

CLUMPAK main pipeline - Job 1746012422 summary

Major modes for the uploaded data:

K=2

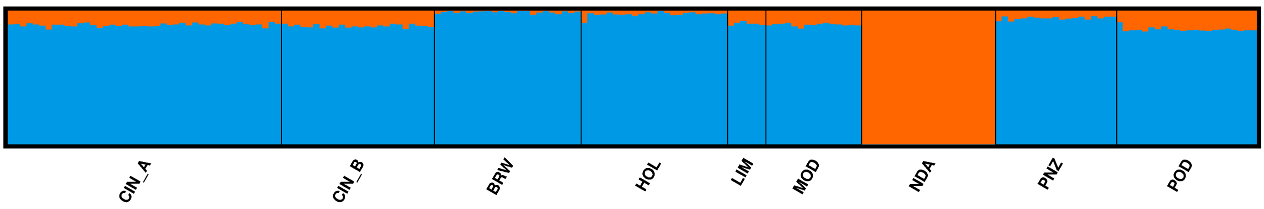

K=3

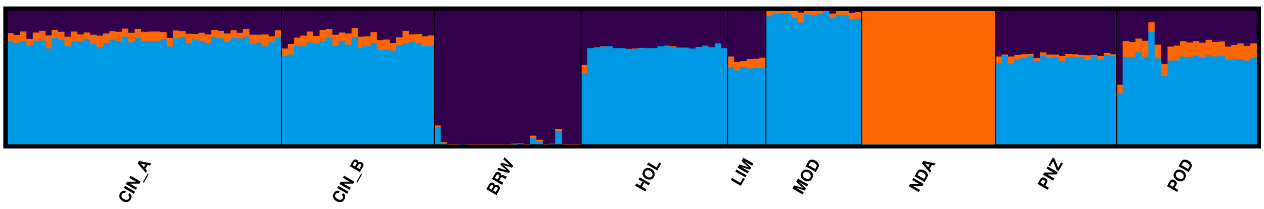

K=4

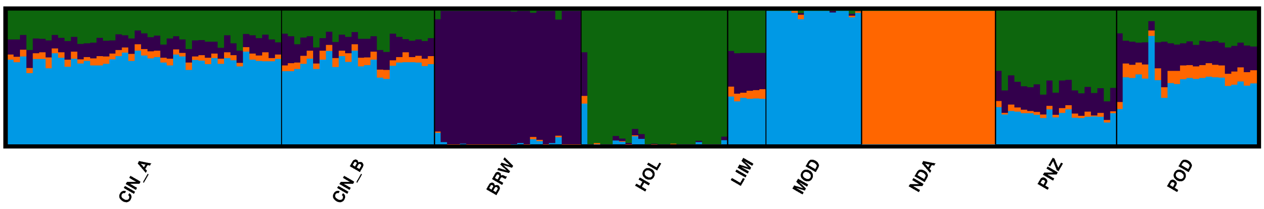

K=5

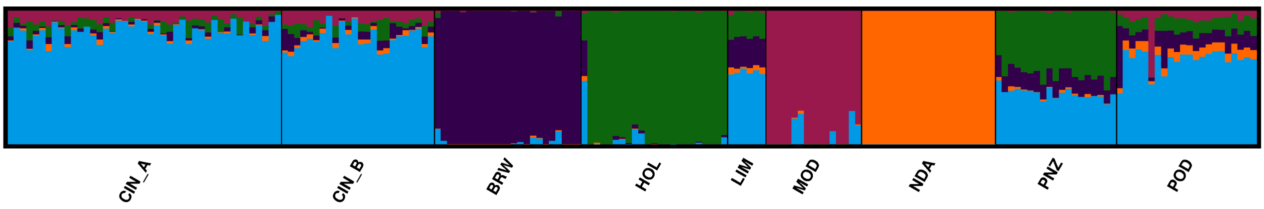

K=6

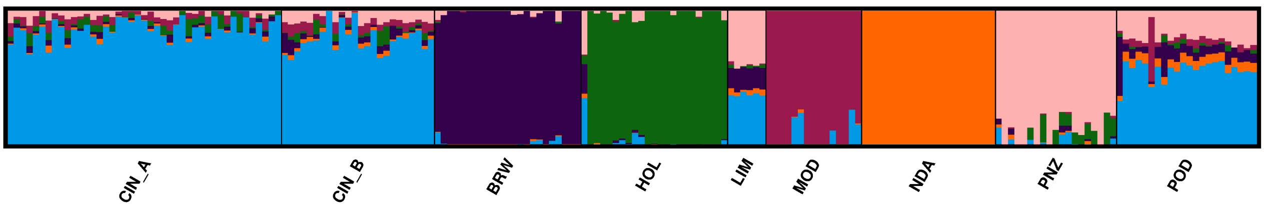

K=7

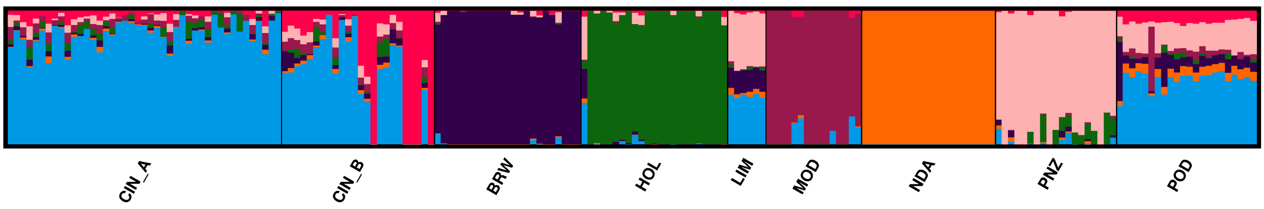

K=8

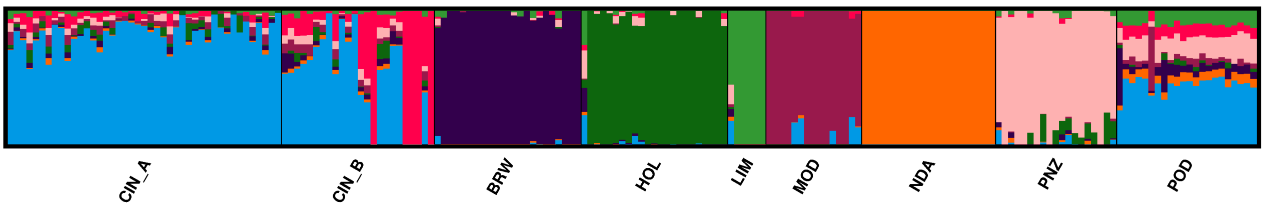

Minor modes for the uploaded data:

Division of runs by mode:

K=2 1/1
K=3 1/1
K=4 1/1
K=5 1/1
K=6 1/1
K=7 1/1
K=8 1/1
