# Supplementary material for: Uncovering the architecture of production-driven introgression in Cinisara cattle breed
Source: BMC Genom Data. 2025 Jul 11;26:47. doi: 10.1186/s12863-025-01337-y (PMC12247468; doi:10.1186/s12863-025-01337-y)
Supplement: Supplementary file 5 — Additional file 5. Local ancestry inferenceconsidering three reference populations: POD as a representative of the ancestral Podolian background, and HOL and BRW that were identified by the former gene flow analyses as candidate sources of introgression. Each Cinisara populationwas individually tested as target population. [file 12863_2025_1337_MOESM5_ESM.pdf]

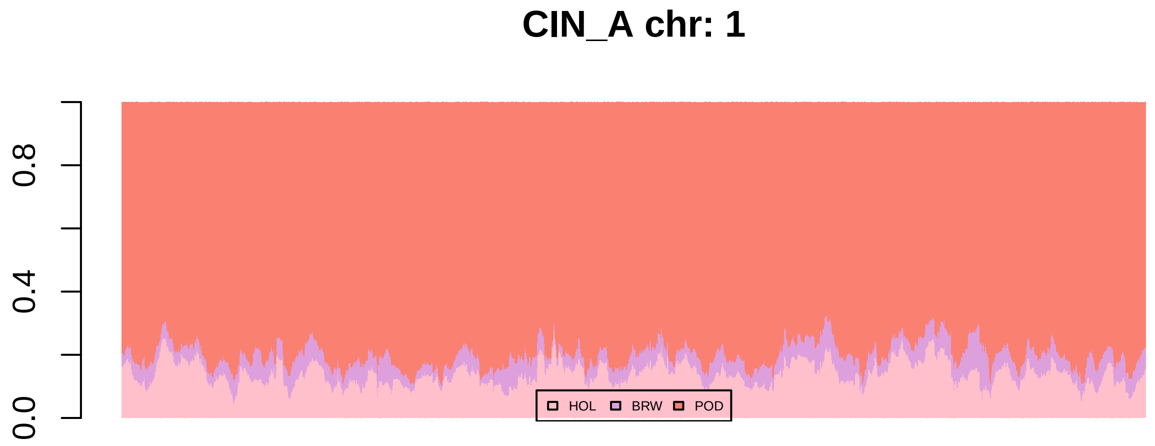
CIN_A chr: 1
0.0
0.4
0.8
HOL
BRW
POD

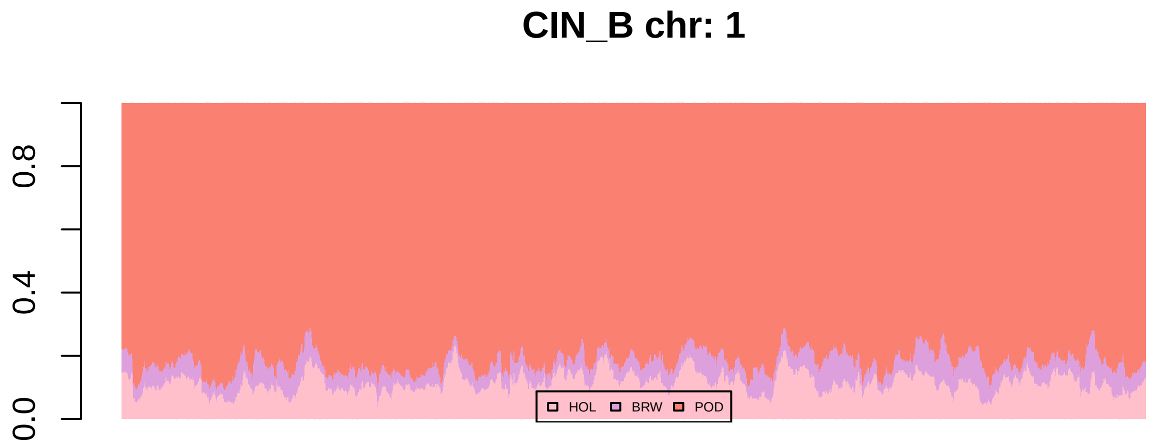
CIN_B chr: 1
0.0
0.4
0.8
HOL
BRW
POD

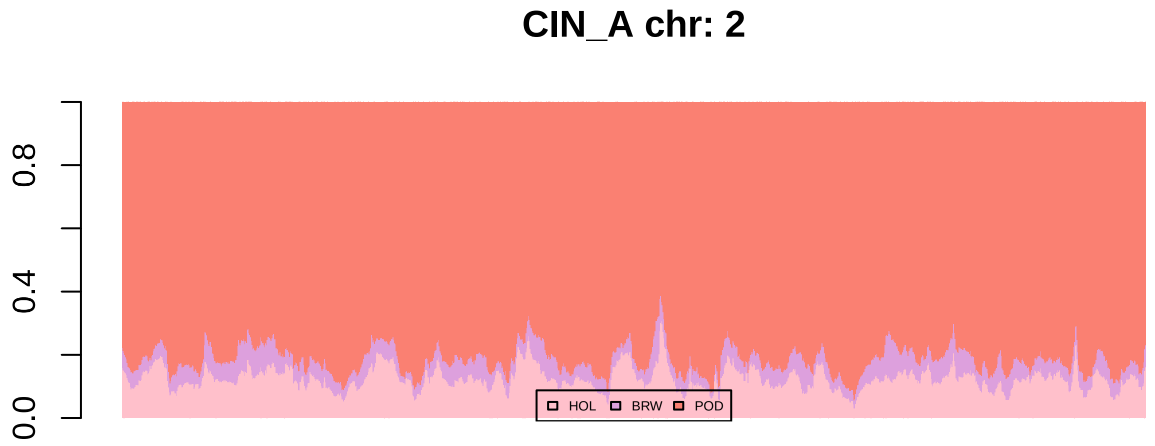

CIN_A chr: 2
0.0
0.4
0.8
HOL
BRW
POD


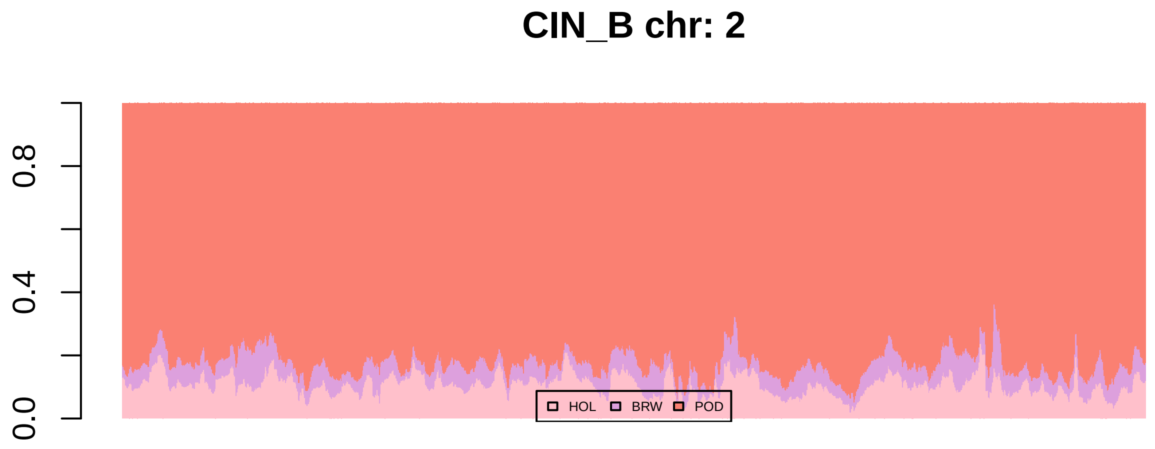

CIN_B chr: 2
0.0
0.4
0.8
HOL
BRW
POD

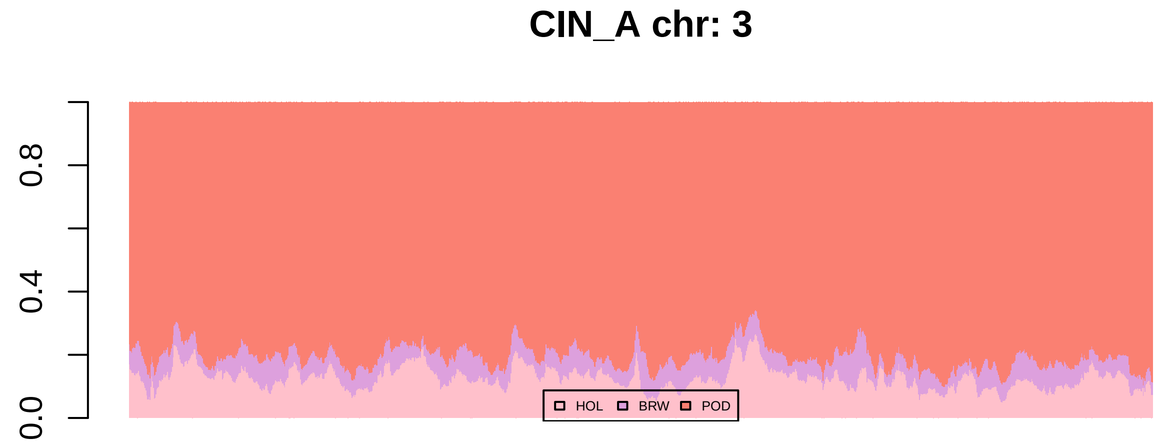
CIN_A chr: 3
0.0
0.4
0.8
HOL
BRW
POD

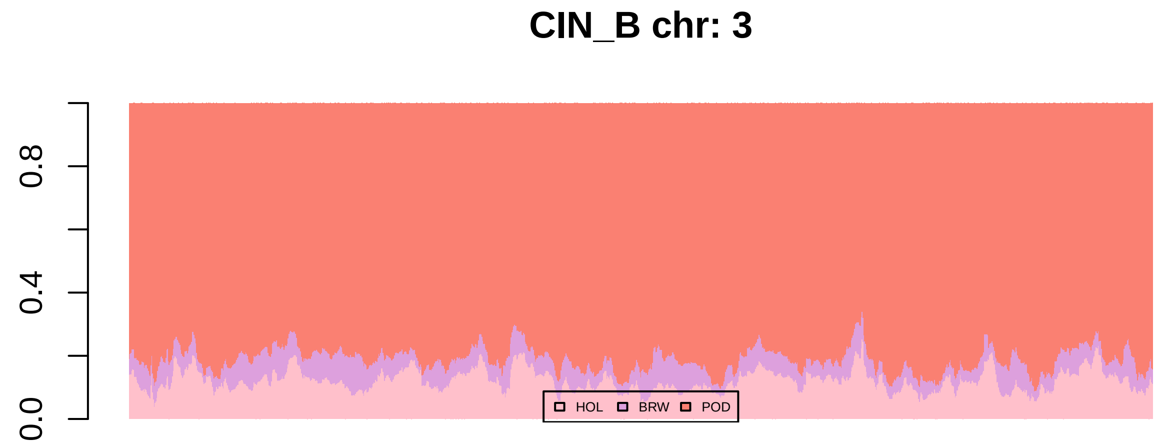
CIN_B chr: 3
0.0
0.4
0.8
HOL
BRW
POD

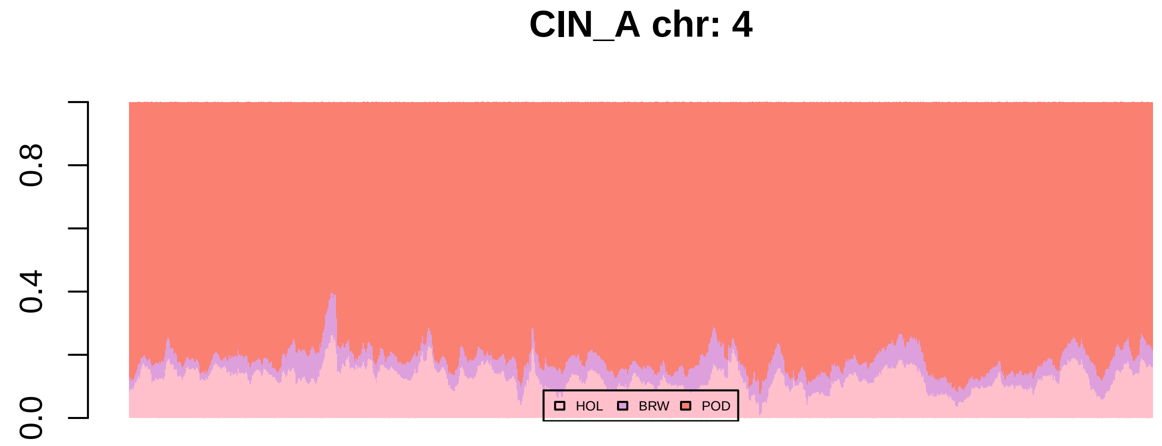
CIN_A chr: 4
0.0
0.4
0.8
HOL
BRW
POD

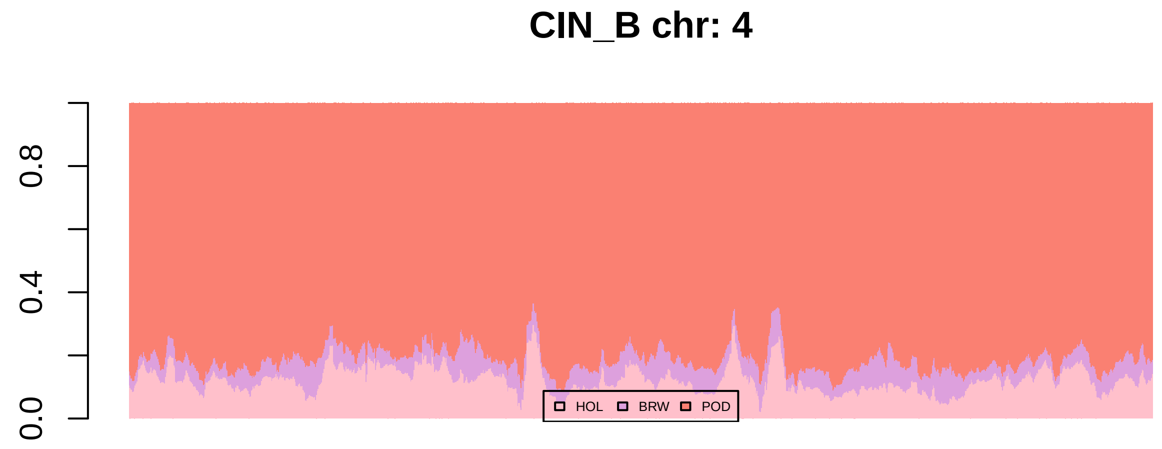
CIN_B chr: 4
0.0
0.4
0.8
HOL
BRW
POD

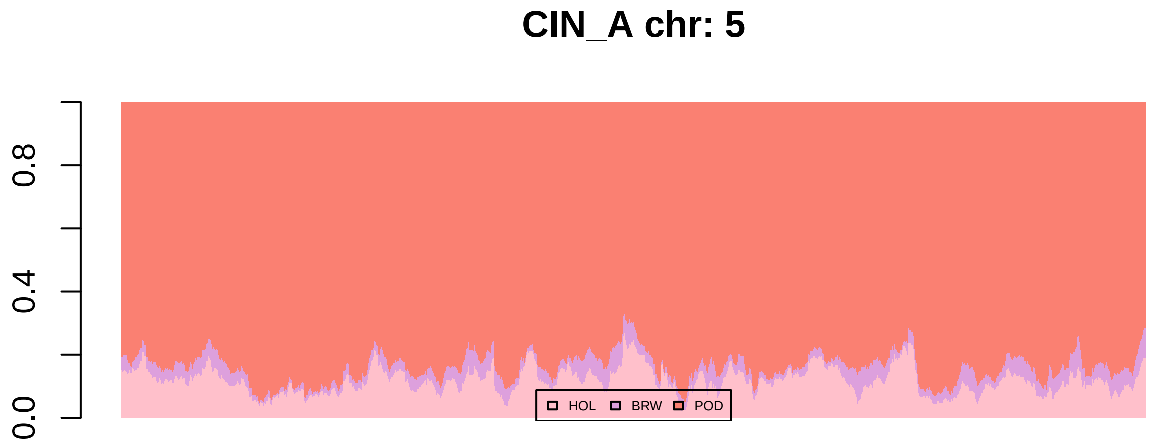
CIN_A chr: 5
0.0
0.4
0.8
HOL
BRW
POD

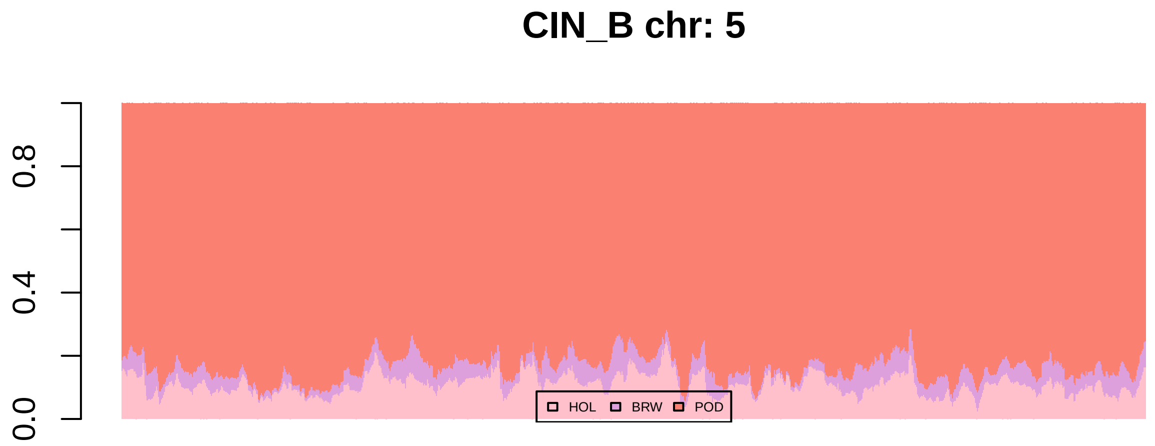
CIN_B chr: 5
0.0
0.4
0.8
HOL
BRW
POD

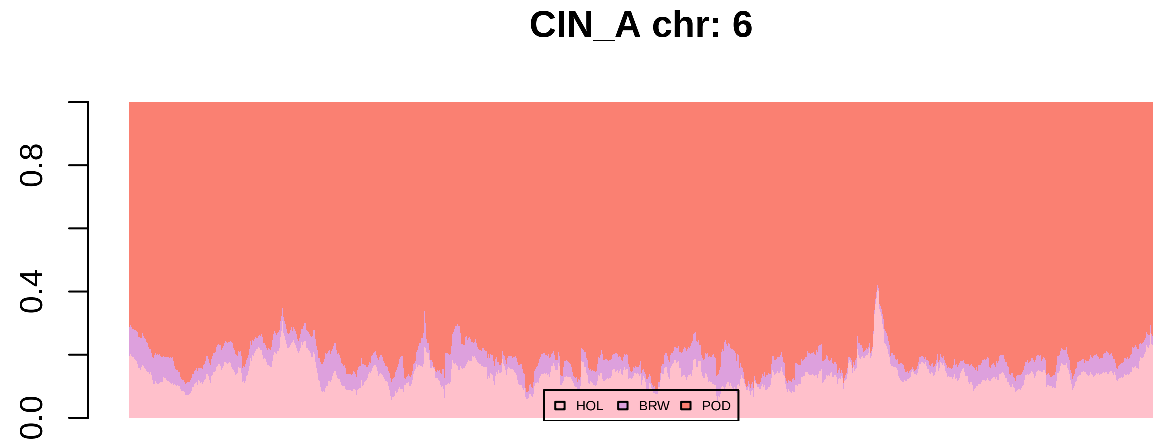
CIN_A chr: 6
0.0
0.4
0.8
HOL
BRW
POD

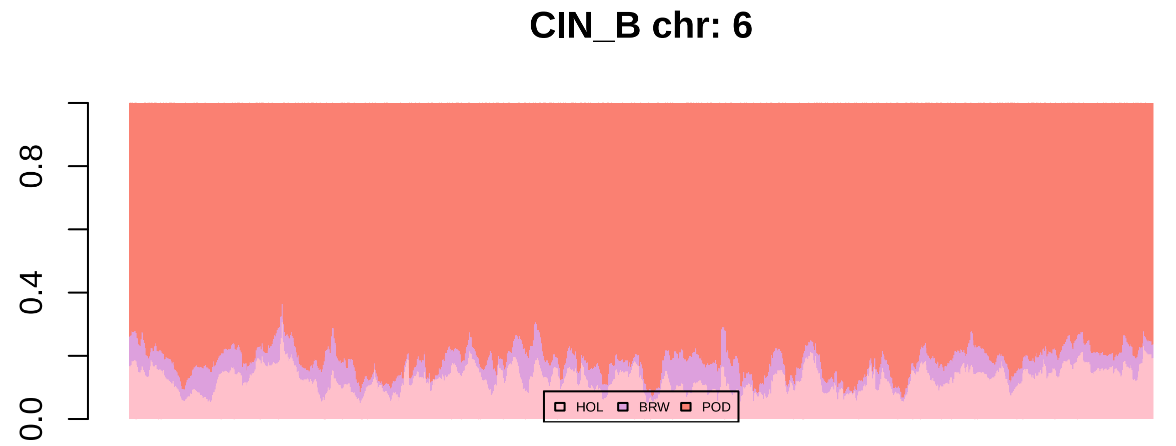
CIN_B chr: 6
0.0
0.4
0.8
HOL
BRW
POD

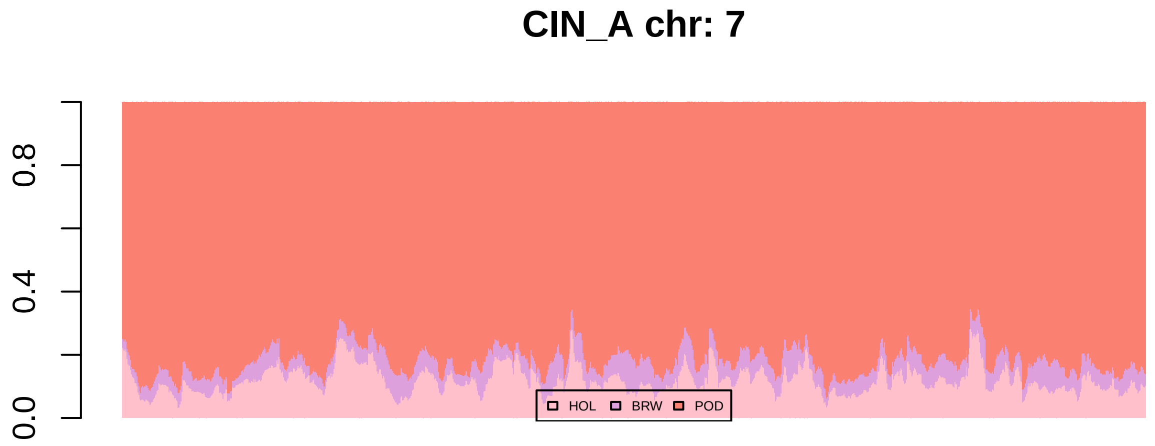
CIN_A chr: 7
0.0
0.4
0.8
HOL
BRW
POD

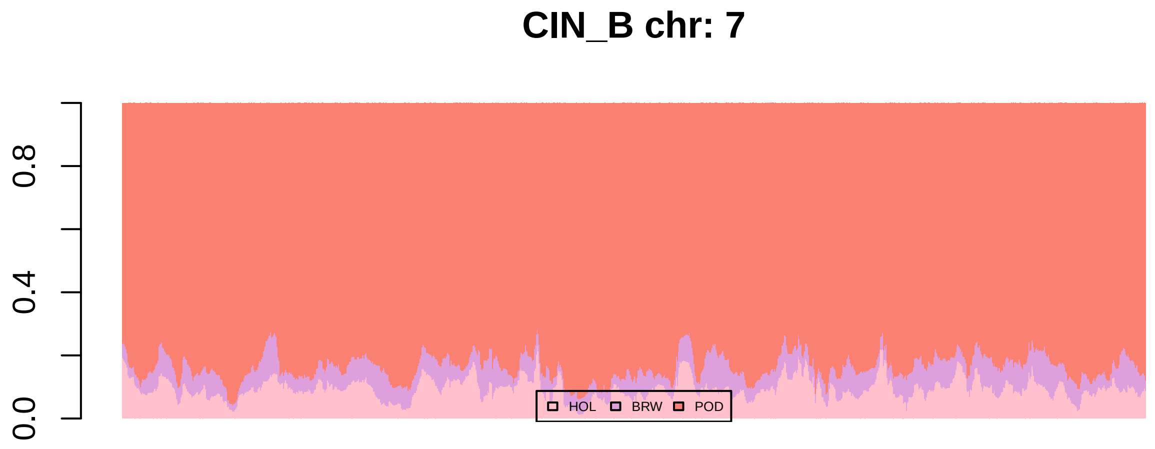
CIN_B chr: 7
0.0
0.4
0.8
HOL
BRW
POD

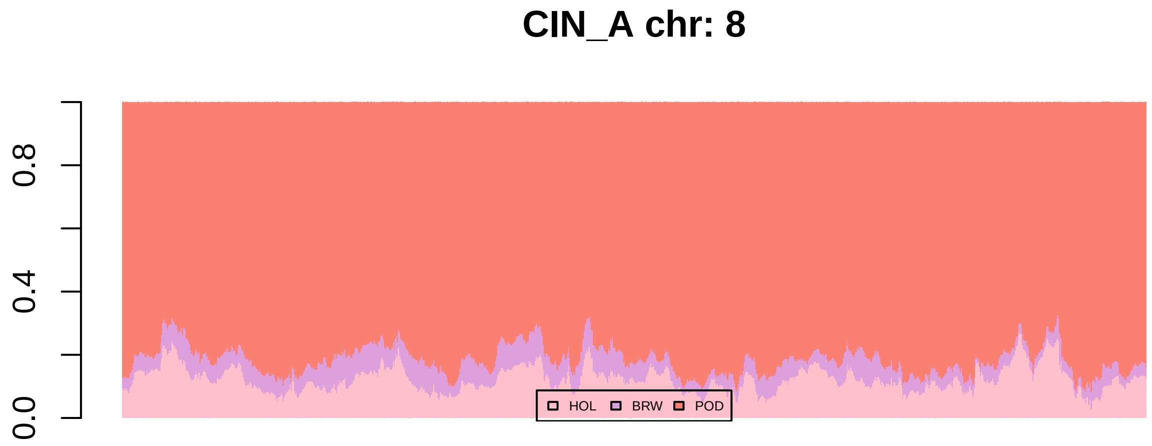
CIN_A chr: 8
0.0
0.4
0.8
HOL
BRW
POD

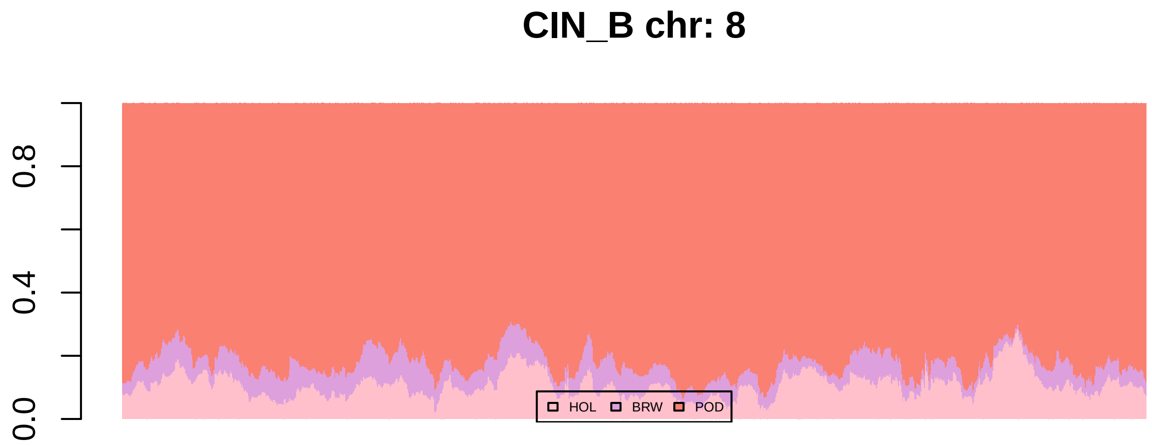
CIN_B chr: 8
0.0
0.4
0.8
HOL
BRW
POD

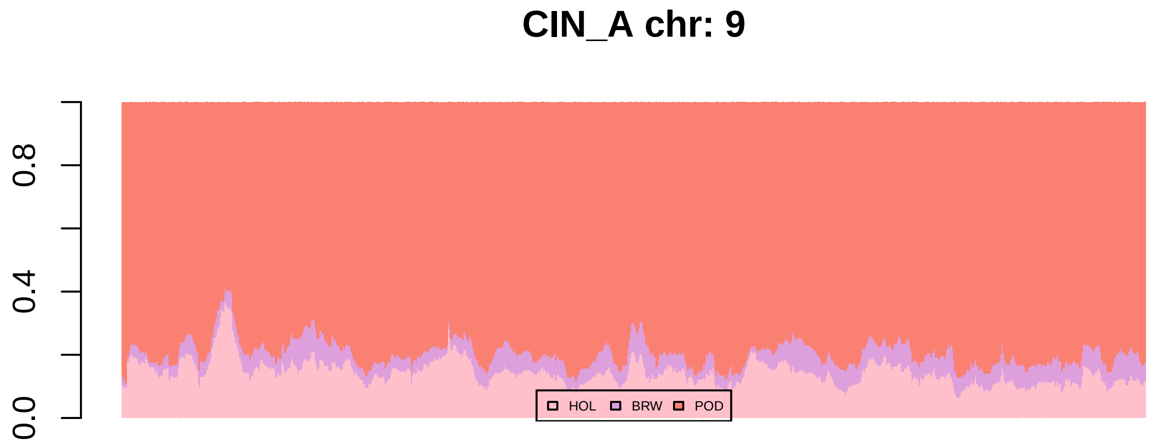
CIN_A chr: 9
0.0
0.4
0.8
HOL
BRW
POD

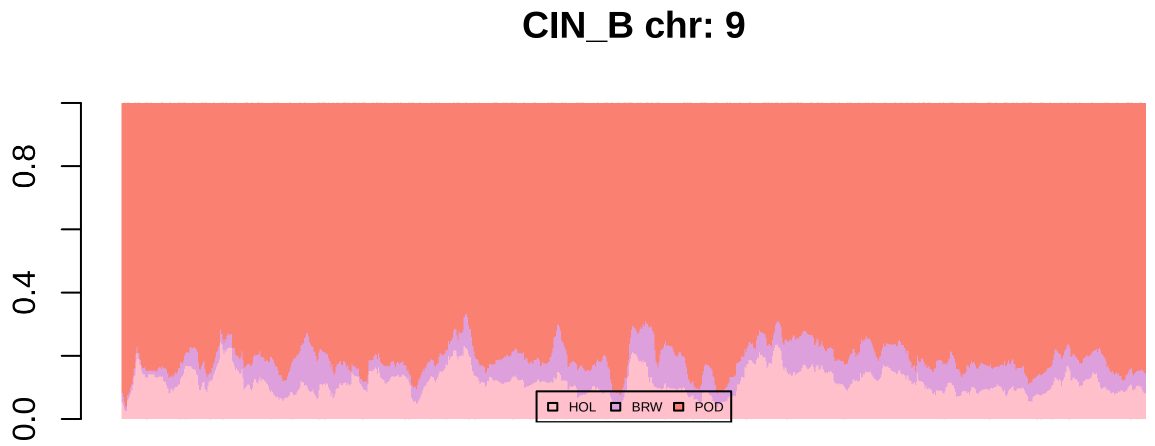
CIN_B chr: 9
0.0
0.4
0.8
HOL
BRW
POD

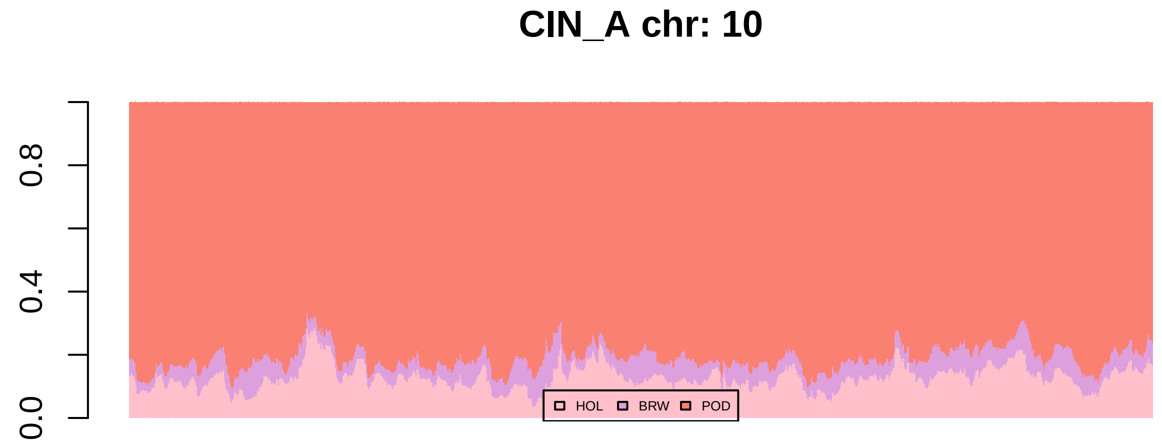

CIN_A chr: 10
0.0
0.4
0.8
HOL
BRW
POD


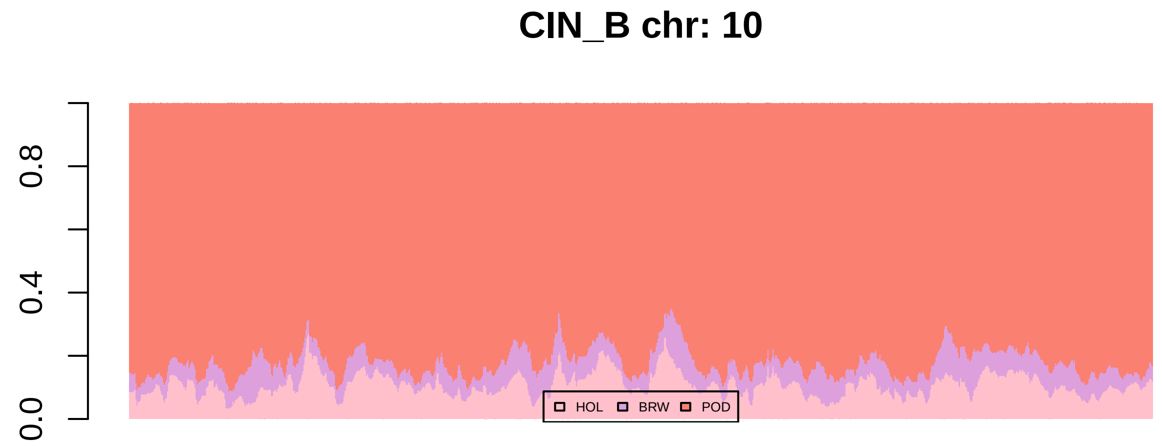

CIN_B chr: 10
0.0
0.4
0.8
HOL
BRW
POD

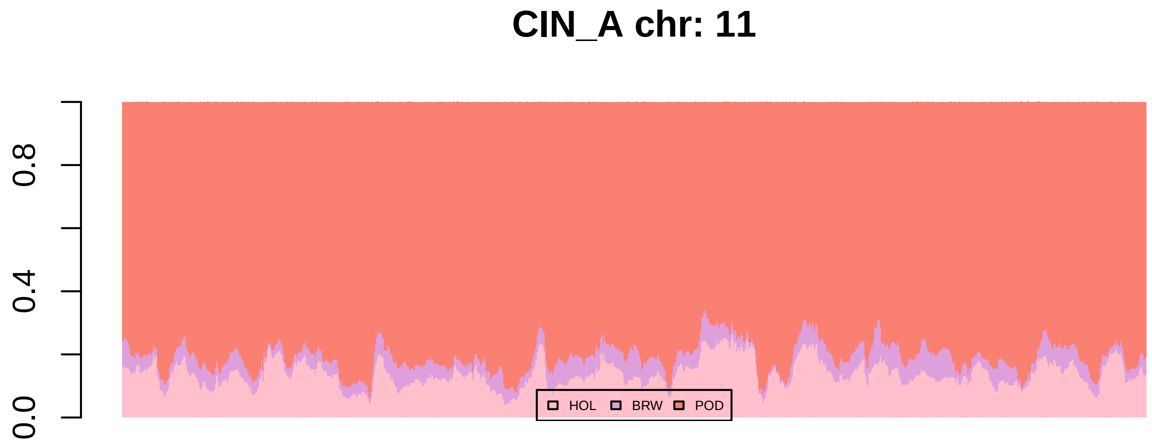
CIN_A chr: 11
0.0
0.4
0.8
HOL
BRW
POD

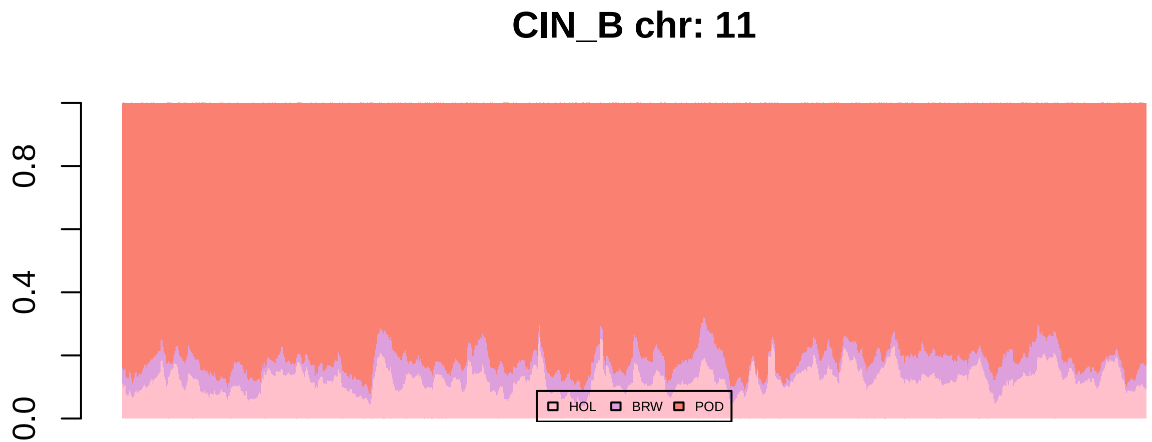
CIN_B chr: 11
0.0
0.4
0.8
HOL
BRW
POD

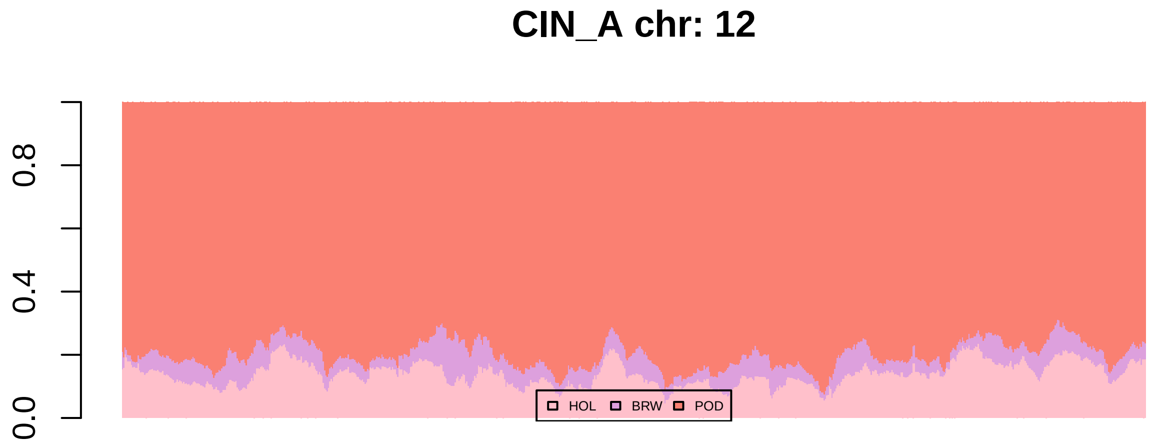
CIN_A chr: 12
0.0
0.4
0.8
HOL
BRW
POD

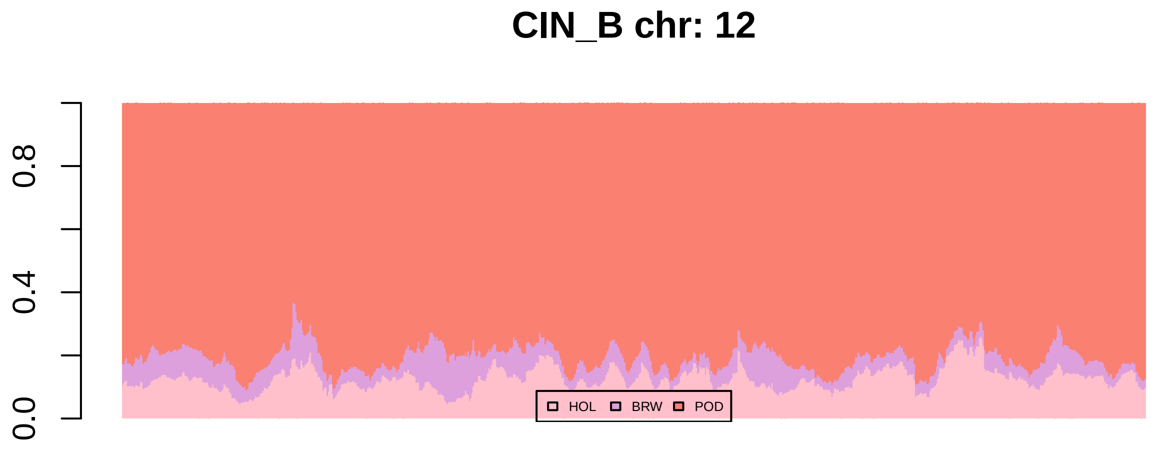
CIN_B chr: 12
0.0
0.4
0.8
HOL
BRW
POD

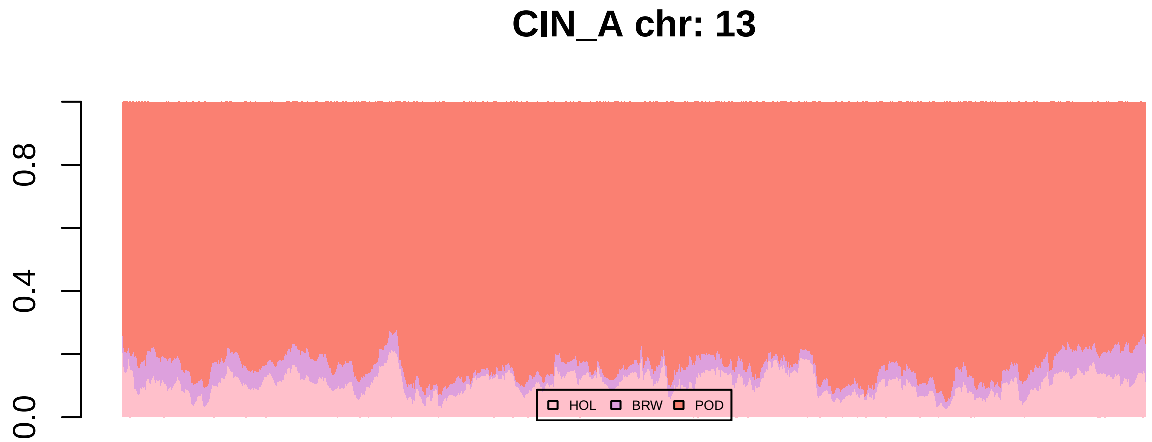
CIN_A chr: 13
0.0
0.4
0.8
HOL
BRW
POD

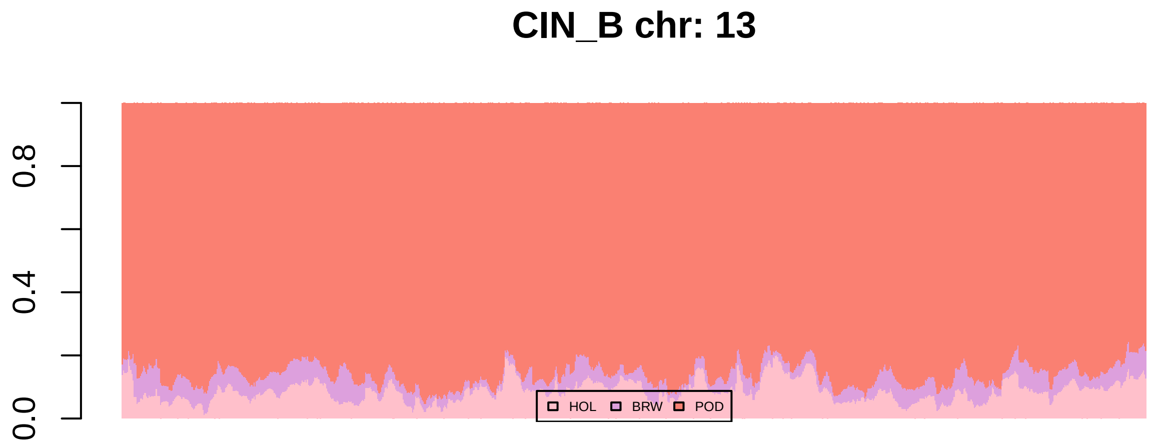
CIN_B chr: 13
0.0
0.4
0.8
HOL
BRW
POD

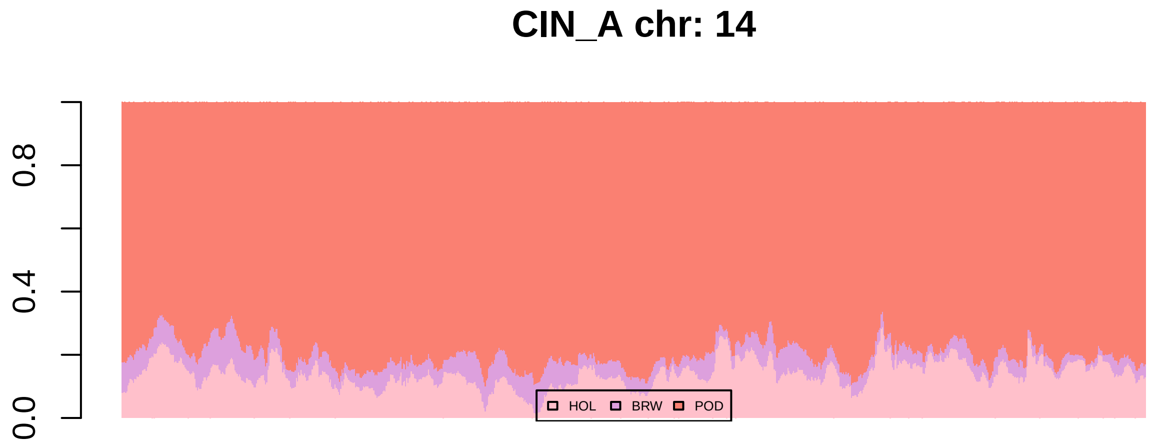
CIN_A chr: 14
0.0
0.4
0.8
HOL
BRW
POD

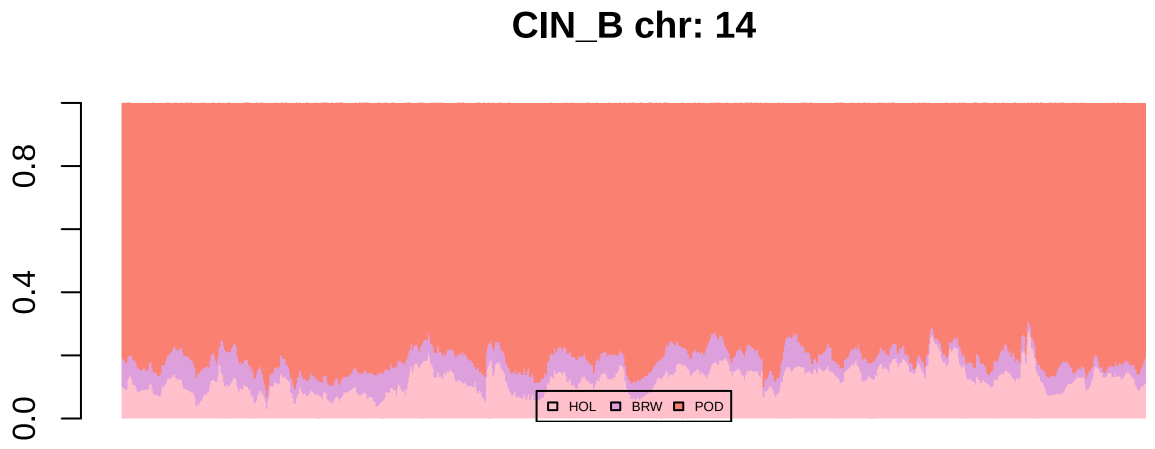
CIN_B chr: 14
0.0
0.4
0.8
HOL
BRW
POD

**CIN_A chr: 15**

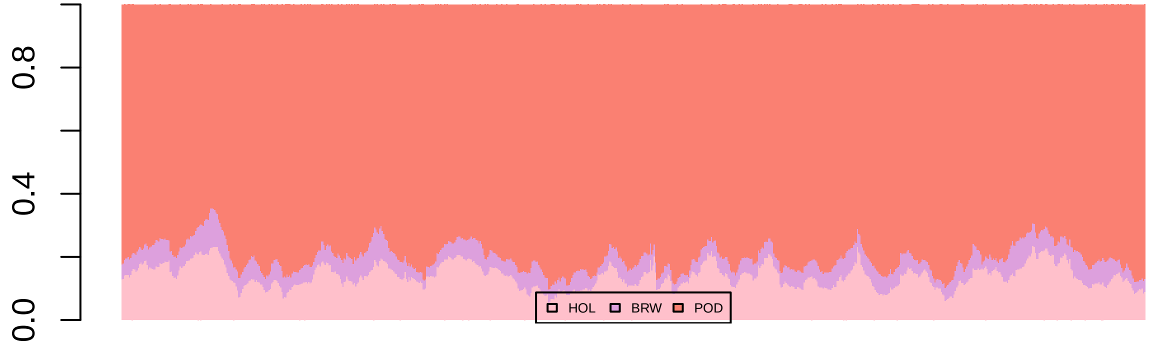


**CIN_B chr: 15**

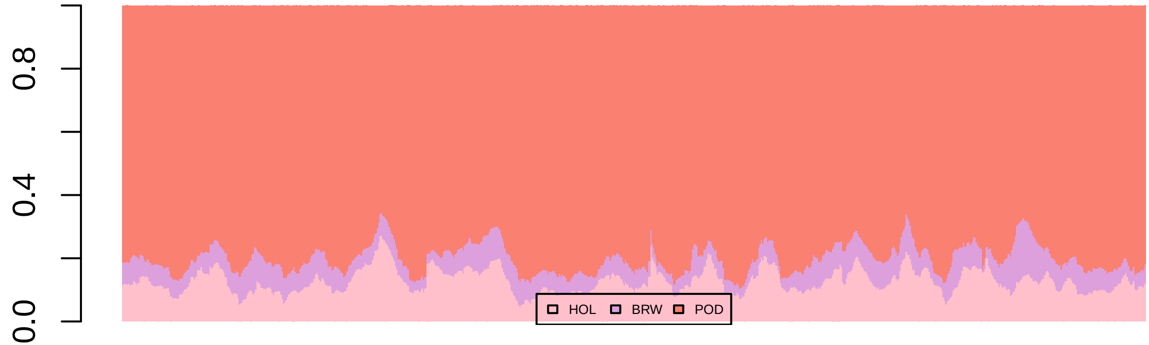

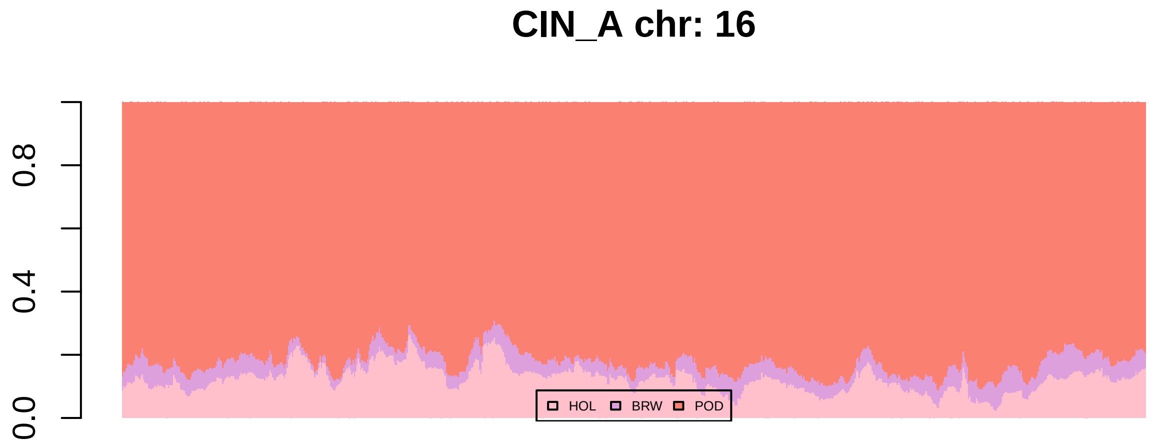

CIN_A chr: 16
0.0
0.4
0.8
HOL
BRW
POD


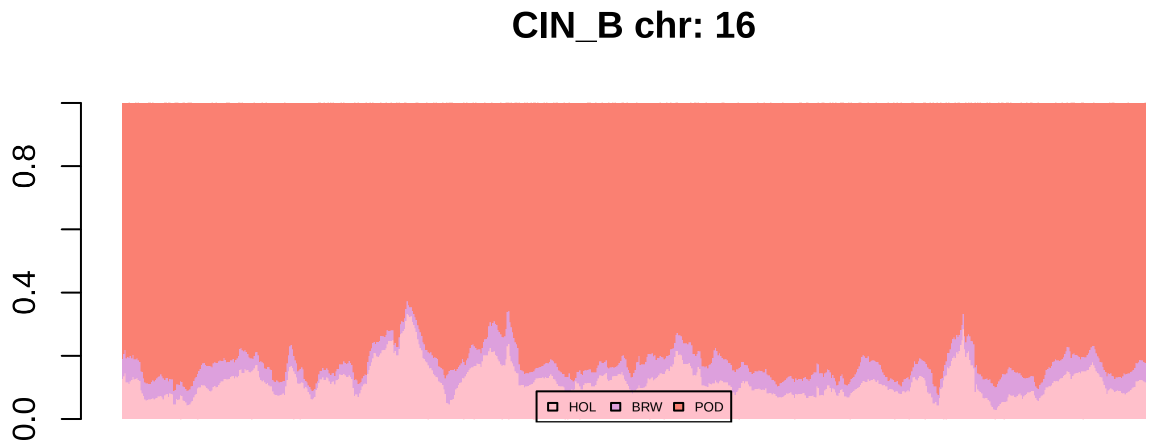

CIN_B chr: 16
0.0
0.4
0.8
HOL
BRW
POD

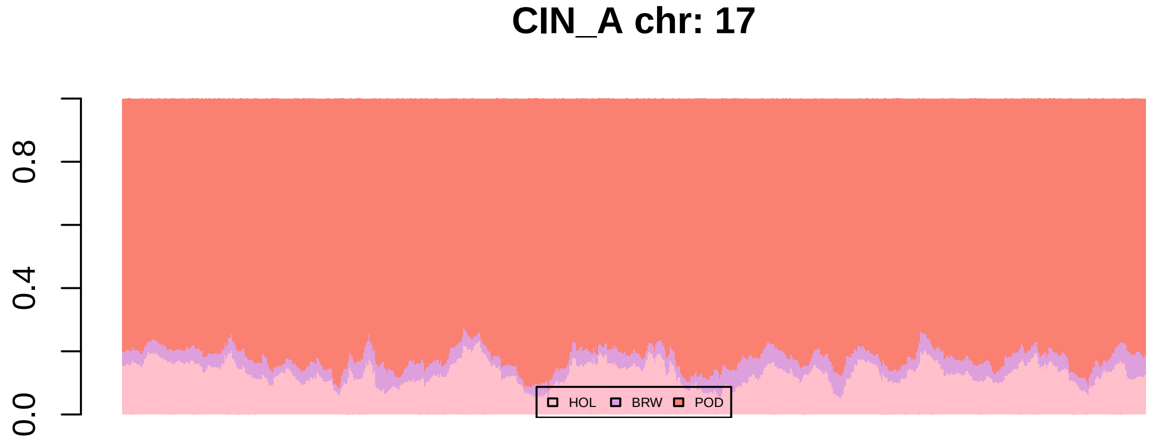
CIN_A chr: 17
0.0
0.4
0.8
HOL
BRW
POD

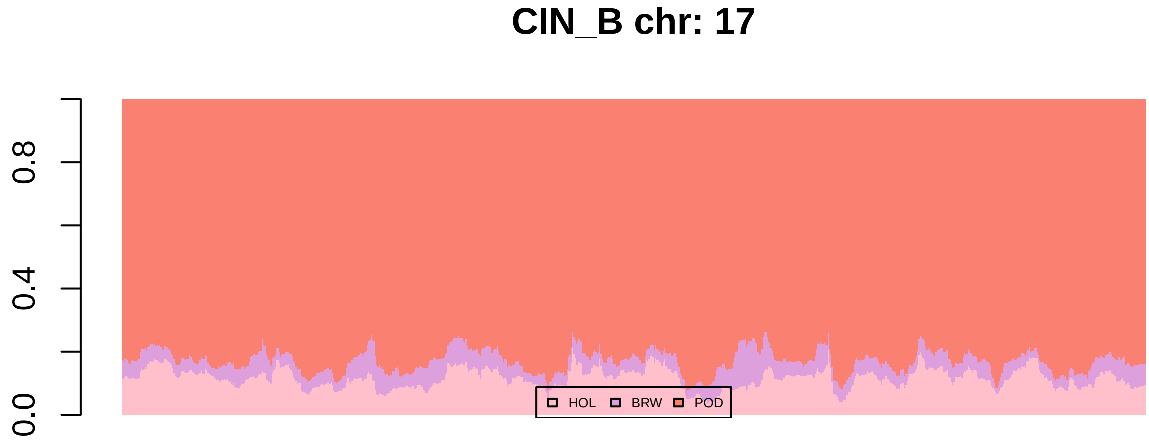
CIN_B chr: 17
0.0
0.4
0.8
HOL
BRW
POD

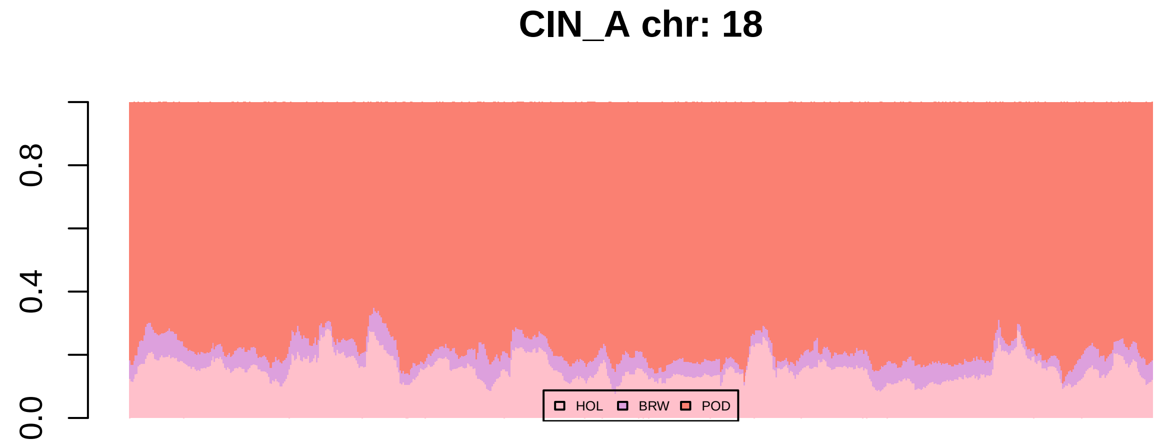
CIN_A chr: 18
0.0
0.4
0.8
HOL
BRW
POD

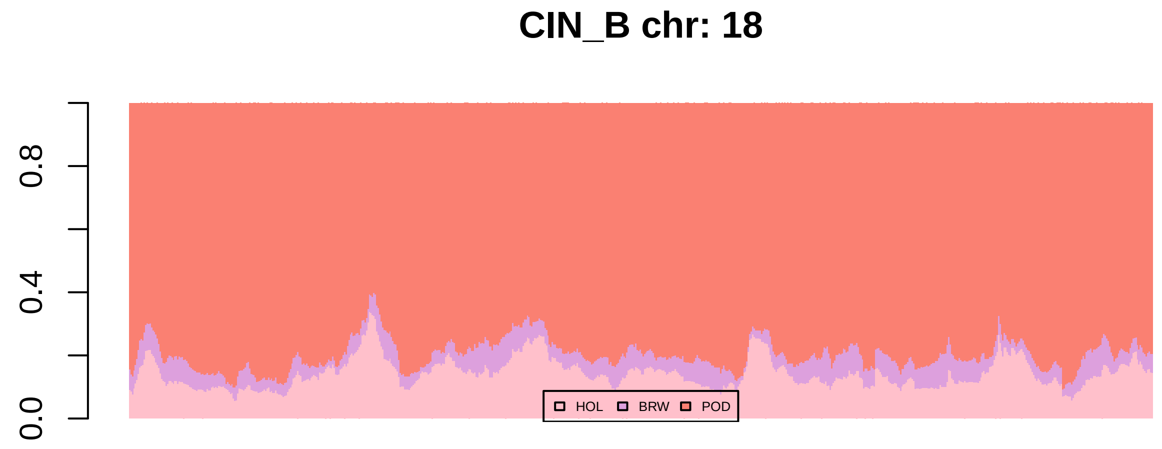
CIN_B chr: 18
0.0
0.4
0.8
HOL
BRW
POD

## CIN_A chr: 19

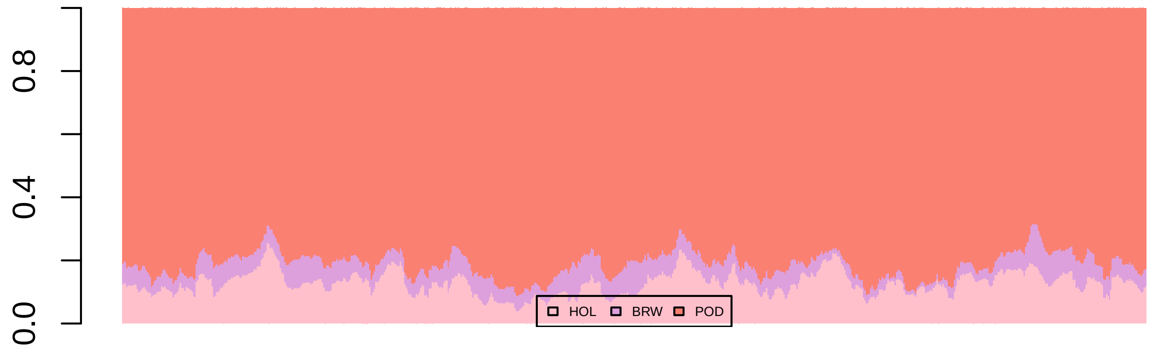


## CIN_B chr: 19

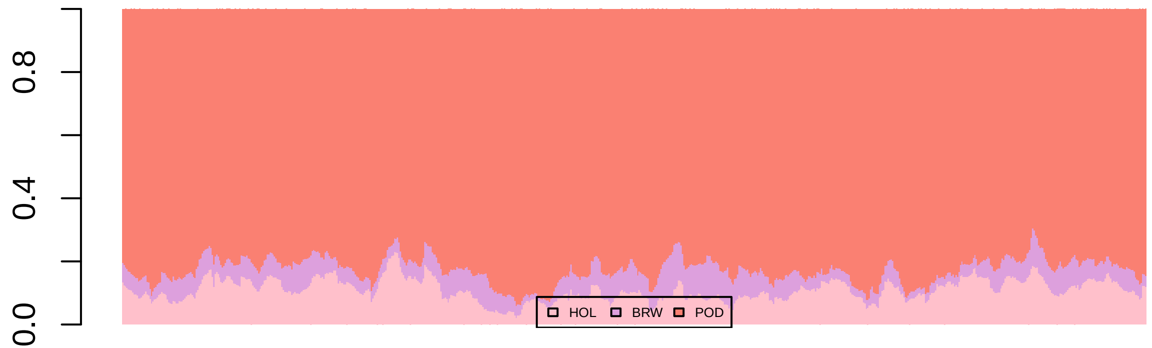

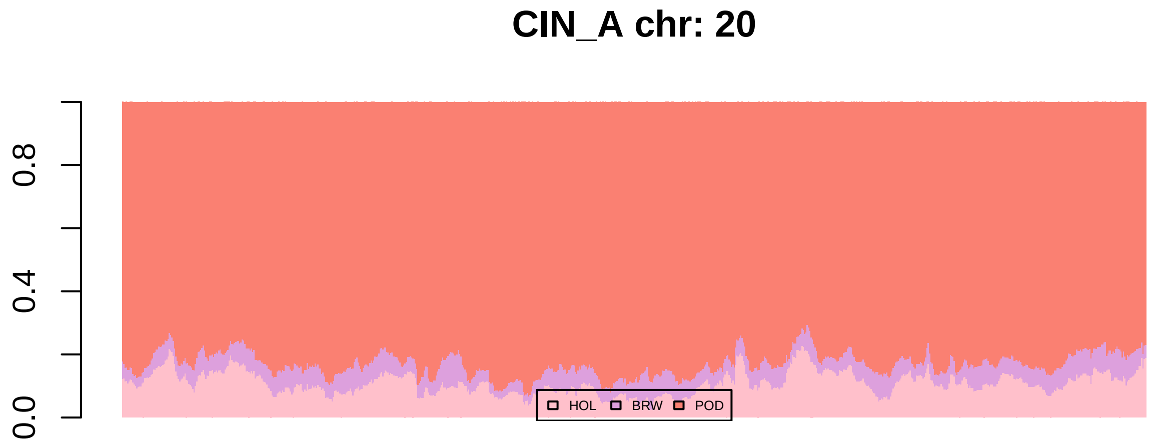
CIN_A chr: 20
0.0
0.4
0.8
HOL
BRW
POD

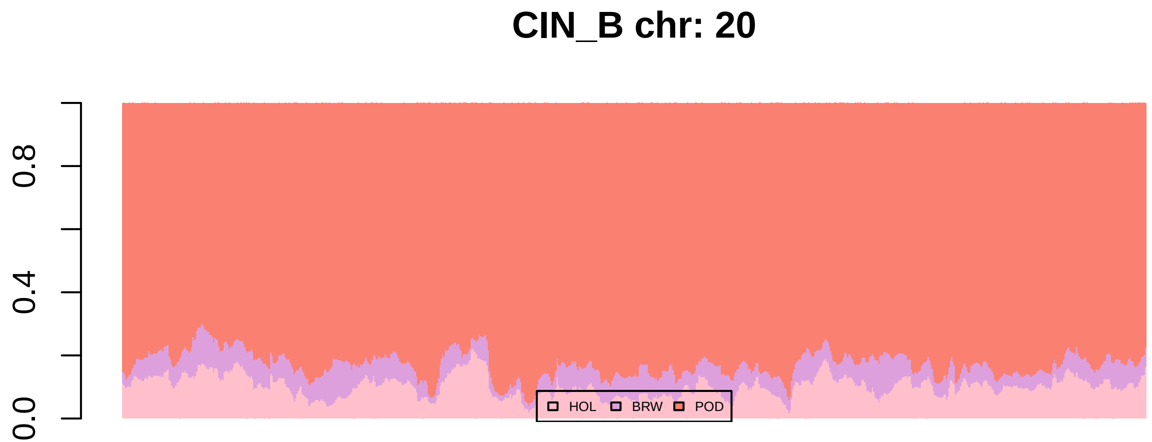
CIN_B chr: 20
0.0
0.4
0.8
HOL
BRW
POD

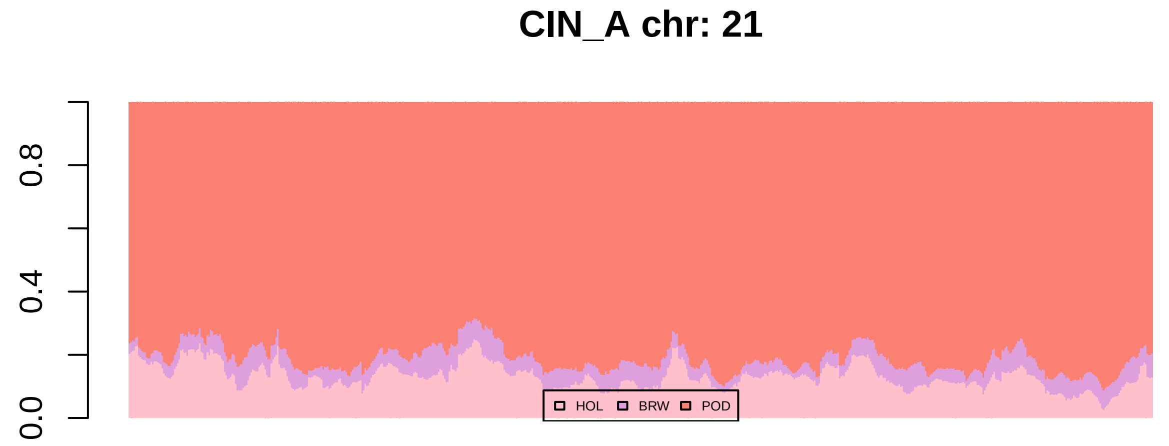
CIN_A chr: 21
0.0
0.4
0.8
HOL
BRW
POD

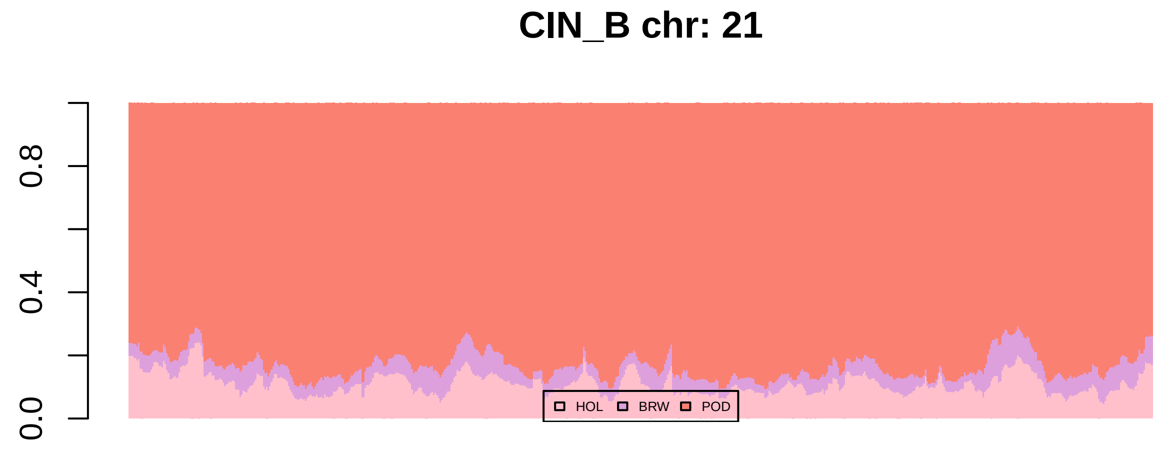
CIN_B chr: 21
0.0
0.4
0.8
HOL
BRW
POD

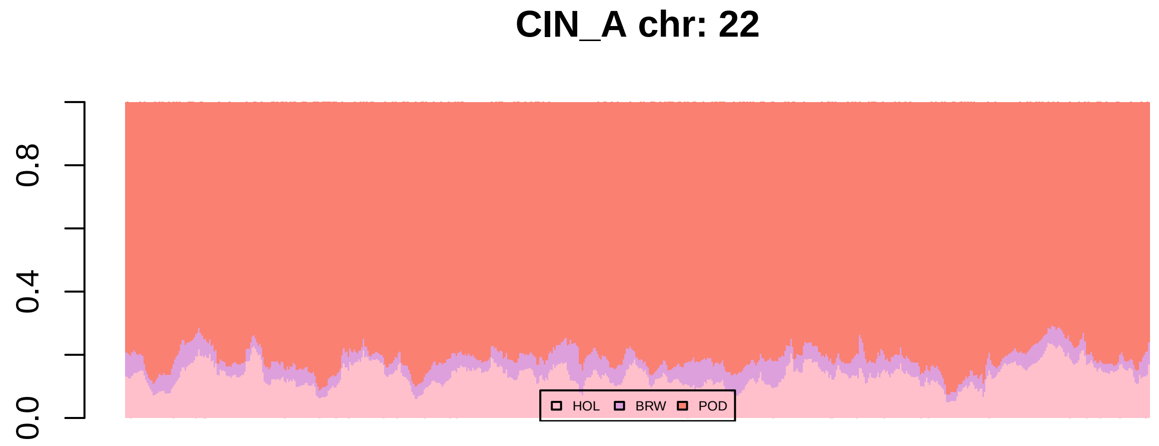

CIN_A chr: 22
0.0
0.4
0.8
HOL
BRW
POD


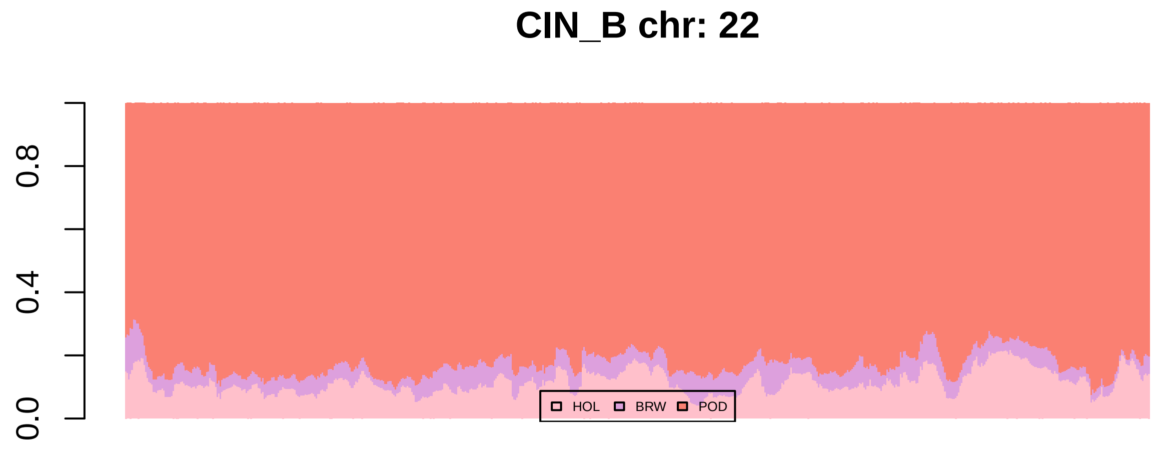

CIN_B chr: 22
0.0
0.4
0.8
HOL
BRW
POD

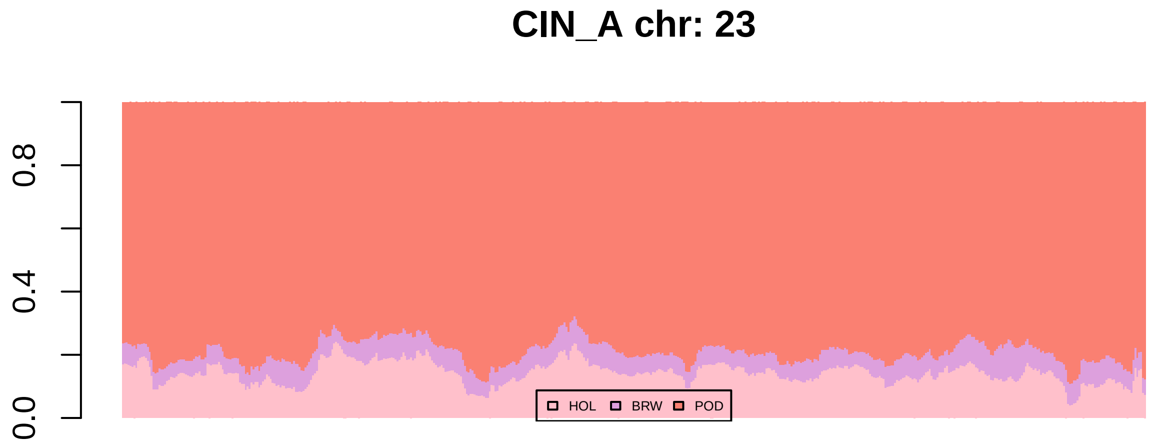
CIN_A chr: 23
0.0
0.4
0.8
HOL
BRW
POD

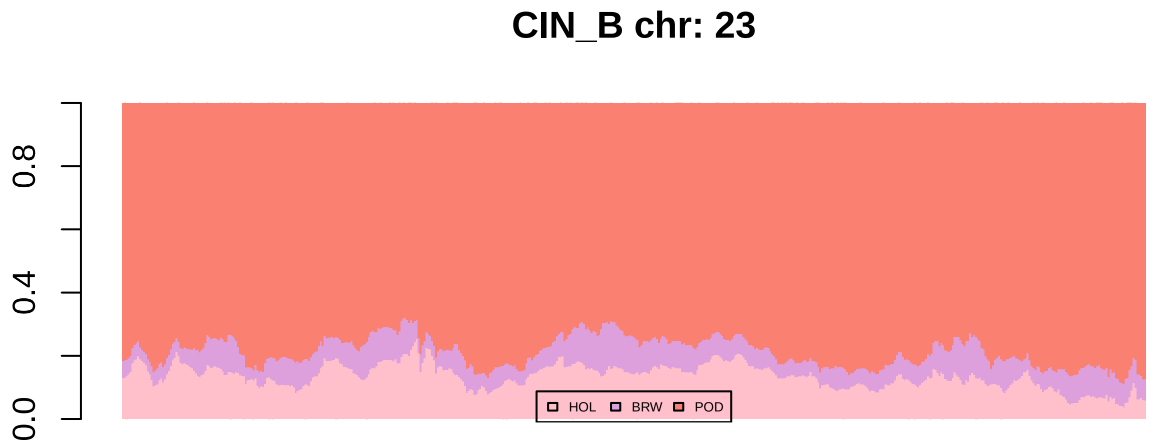
CIN_B chr: 23
0.0
0.4
0.8
HOL
BRW
POD

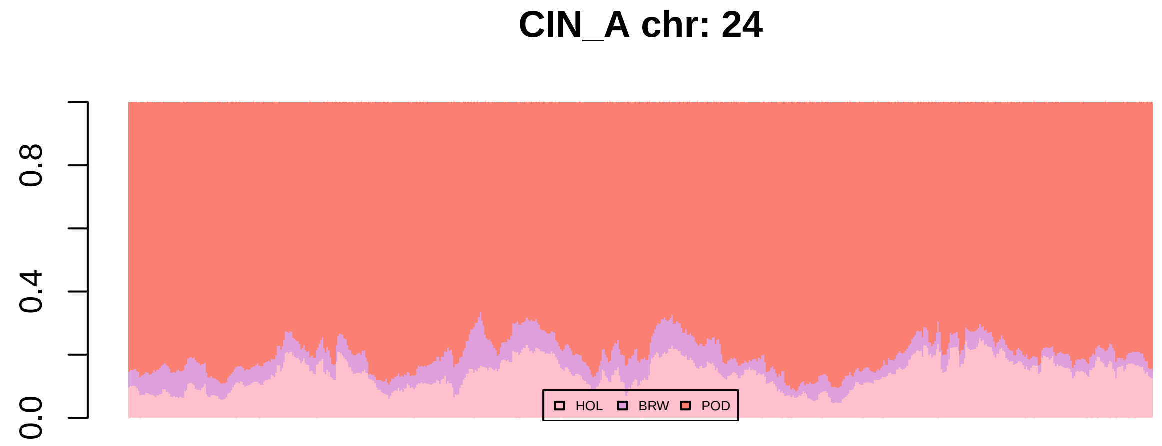
CIN_A chr: 24
0.0
0.4
0.8
HOL
BRW
POD

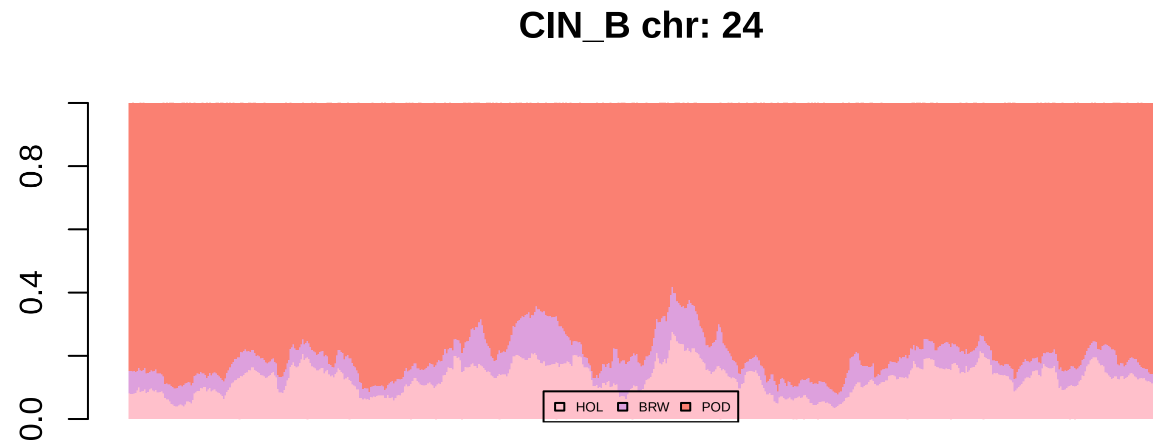
CIN_B chr: 24
0.0
0.4
0.8
HOL
BRW
POD

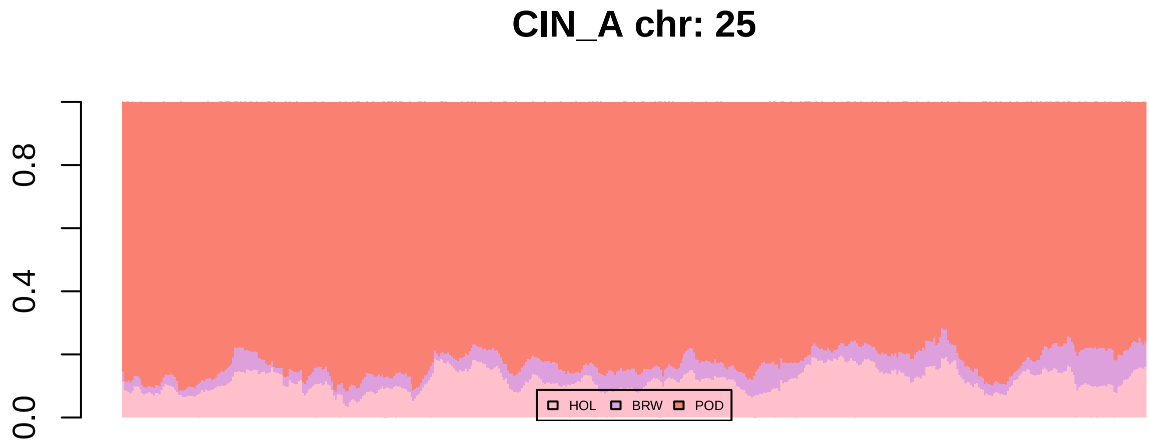
CIN_A chr: 25
0.0
0.4
0.8
HOL
BRW
POD

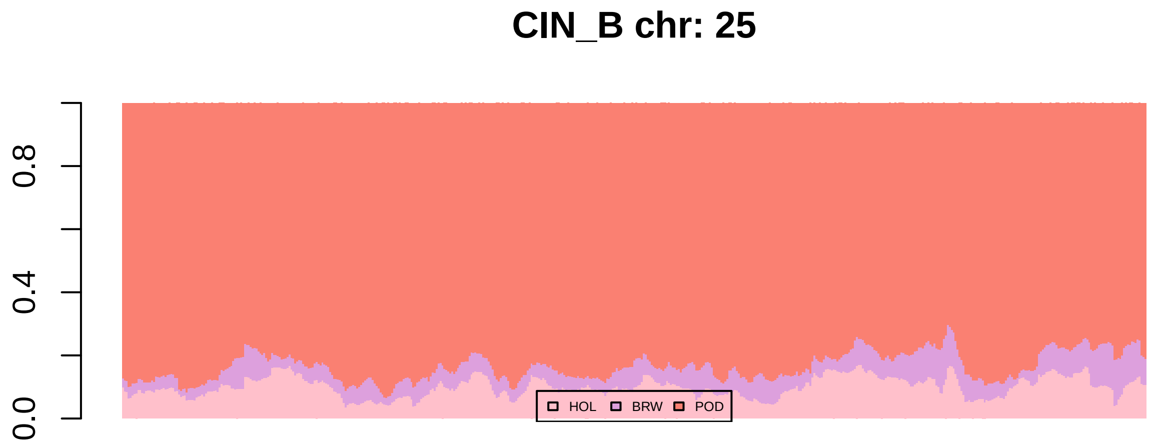
CIN_B chr: 25
0.0
0.4
0.8
HOL
BRW
POD

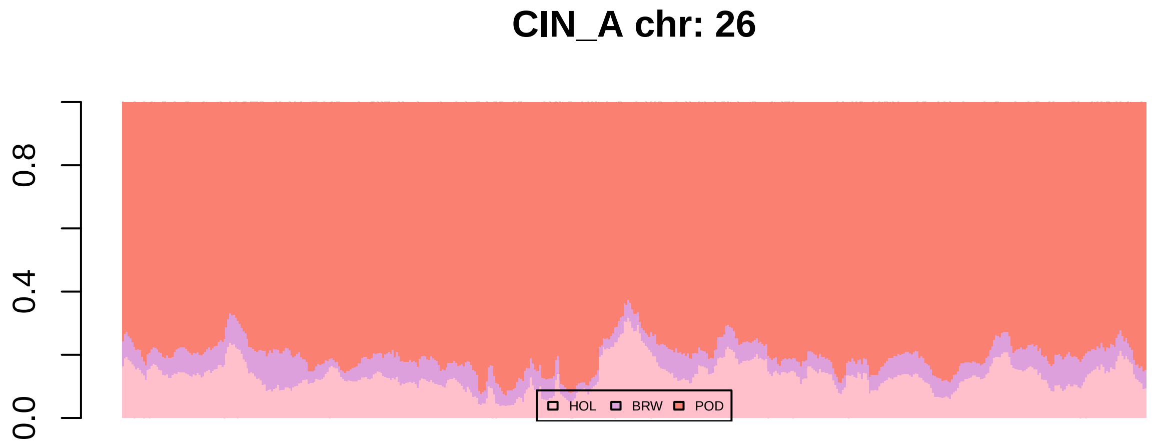
CIN_A chr: 26
0.0
0.4
0.8
HOL
BRW
POD

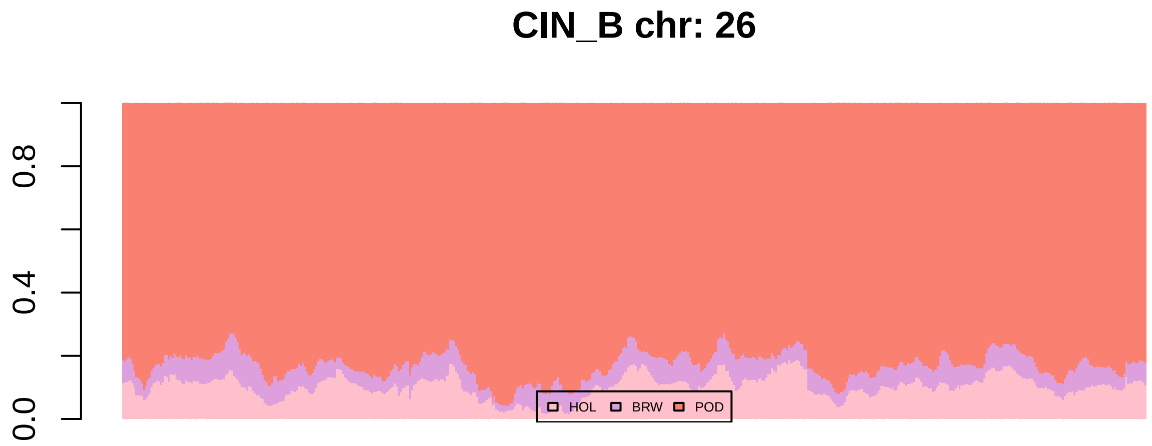
CIN_B chr: 26
0.0
0.4
0.8
HOL
BRW
POD

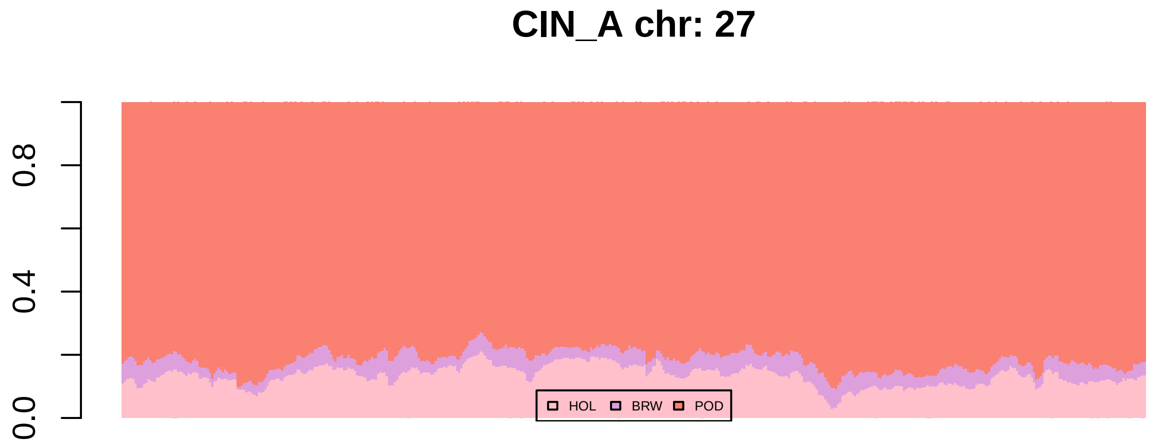
CIN_A chr: 27
0.0
0.4
0.8
HOL
BRW
POD

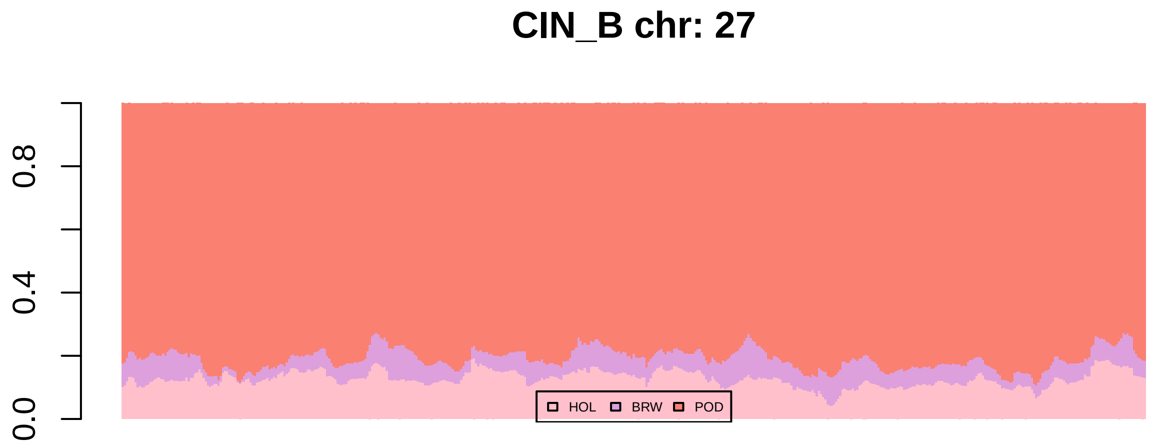
CIN_B chr: 27
0.0
0.4
0.8
HOL
BRW
POD

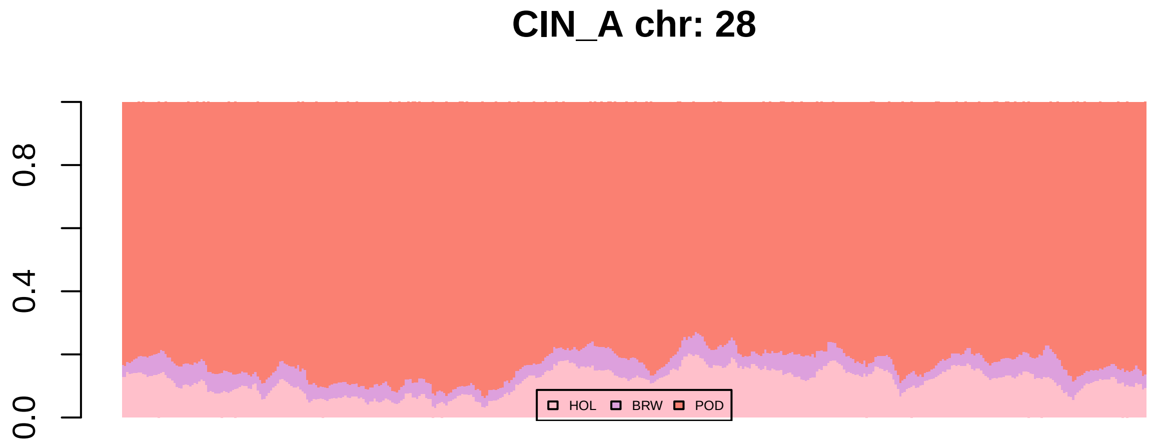
CIN_A chr: 28
0.0
0.4
0.8
HOL
BRW
POD

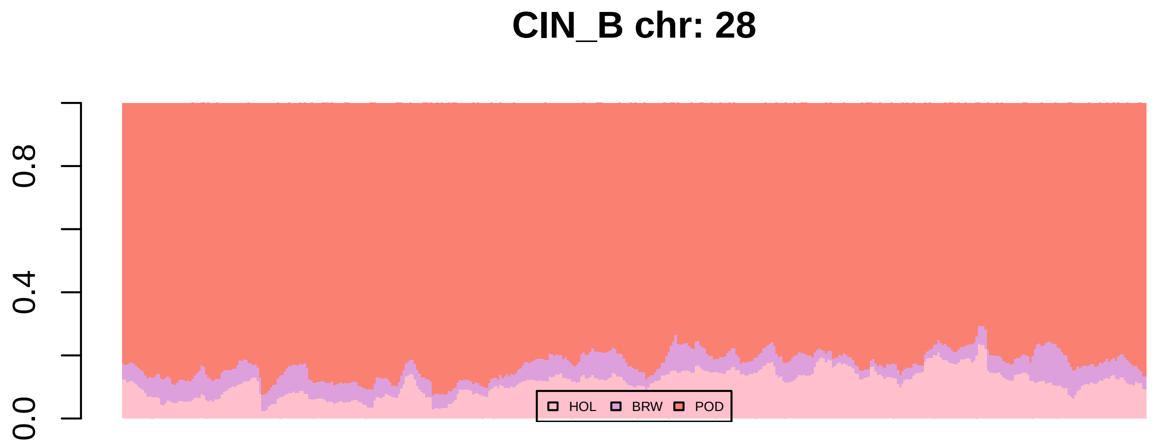
CIN_B chr: 28
0.0
0.4
0.8
HOL
BRW
POD

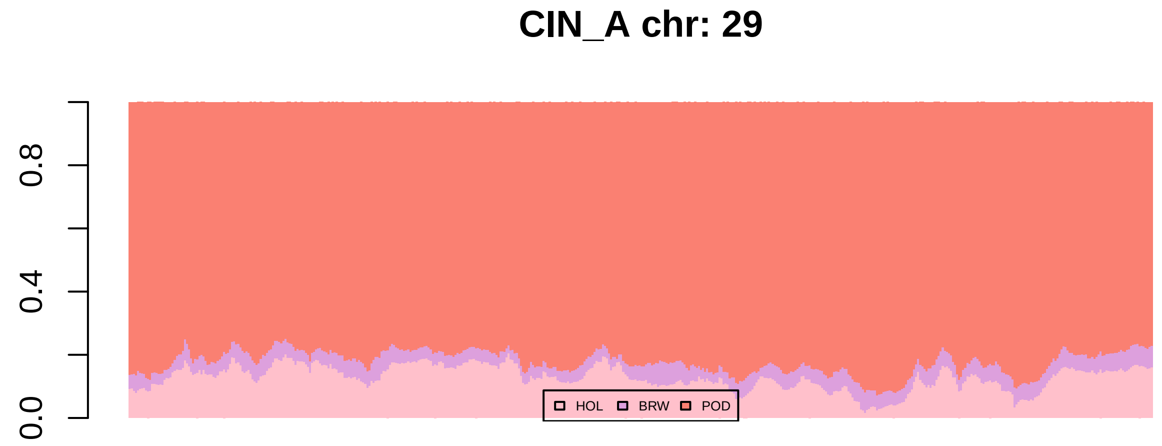
CIN_A chr: 29
0.0
0.4
0.8
HOL
BRW
POD

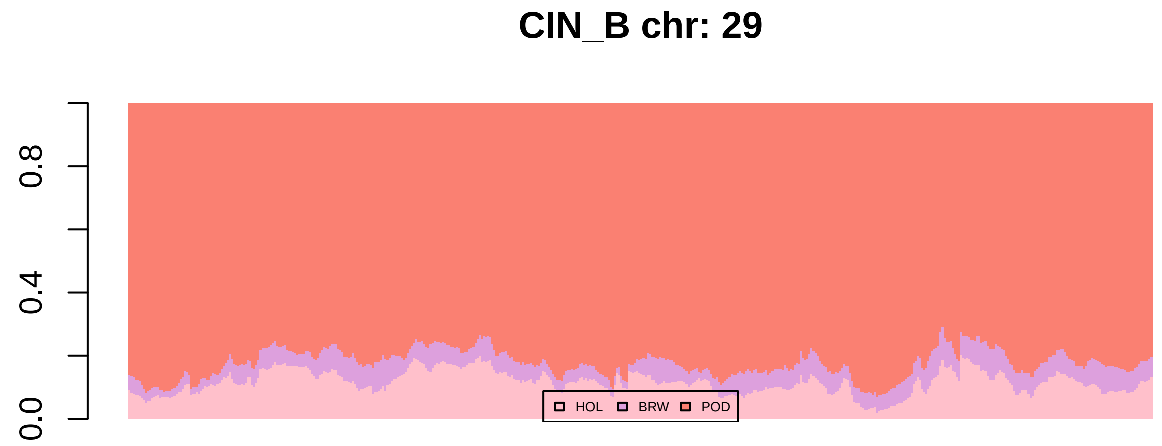
CIN_B chr: 29
0.0
0.4
0.8
HOL
BRW
POD
